# Resilient tree-planting strategies for carbon dioxide removal under compounding climate and economic uncertainties

**DOI:** 10.1073/pnas.2320961122

**Published:** 2025-03-03

**Authors:** Frankie H. T. Cho, Paolo Aglonucci, Ian J. Bateman, Christopher F. Lee, Andrew Lovett, Mattia C. Mancini, Chrysanthi Rapti, Brett H. Day

**Affiliations:** ^a^Land, Environment, Economics and Policy Institute, Department of Economics, University of Exeter, Exeter EX4 4PU, United Kingdom; ^b^Centre for Biodiversity and Conservation Science, University of Queensland, Brisbane QLD 4072, Australia; ^c^School of the Environment, University of Queensland, Brisbane QLD 4072, Australia; ^d^Institute for Sustainable Resources, Bartlett School of Environment, Energy and Resources, University College London, London WC1H 0NN, United Kingdom; ^e^School of Environmental Sciences, University of East Anglia, Norwich NR4 7TJ, United Kingdom

**Keywords:** climate risks, tree planting, carbon dioxide removal, uncertainty, portfolio optimization

## Abstract

To meet decarbonization targets, nations around the globe have made ambitious commitments to expand forested land. Operationalizing these commitments requires choosing a planting strategy: How many trees should be planted, of which species, and where? Given those choices must be made now but have long-term consequences, such decisions are plagued by uncertainty. For example, species that are well suited to present conditions may perform poorly under future climates, yet those future climates are themselves highly uncertain. Using the exemplar of the United Kingdom, a nation committed to achieving net zero emissions by midcentury, we quantify key uncertainties pertaining to coevolving climate and economic conditions and examine how modern methods of decision-making under uncertainty can advise on planting choices. Our analysis reveals that the best planting strategy assuming a “high-emissions” future is radically different to that for a future that remains on a “near-historic” path. Planting for the former while experiencing the latter results in substantial net costs to UK society. Assimilating uncertainty into decision-making identifies planting strategies that diversify risk and significantly reduce the probability of high-cost outcomes. Importantly, our research reveals that the scope for mitigating risk through choice of planting strategy is relatively limited. Despite this persistent risk, we find that tree planting remains a highly cost-effective carbon removal solution when compared to alternative technologies, even when those alternatives are assumed to be riskless.

## Significance

For many countries, large-scale tree planting is a crucial component of their decarbonization plans. This paper positions those decisions in the context of the substantial uncertainties surrounding both future climatic and economic conditions. Using the United Kingdom as an example, this paper finds the following: 1) Nations can expose themselves to substantial cost risk by pursuing planting strategies that ignore uncertainty. 2) Planting strategies that use portfolio approaches to diversify risk can substantially reduce exposure to downside cost extremes. 3) Portfolio approaches can mitigate some risk exposure, but significant cost risks still exist. 4) Despite this persistent risk profile, when compared to projected costs for alternative technologies, tree planting emerges as a highly cost-effective option for carbon dioxide removal.

To honor commitments made under the Paris Agreement and in support of initiatives such as the Bonn Challenge, nations across the world are developing plans to plant trees as a means of removing atmospheric greenhouse gases. The scale of promised planting is substantial. The European Commission has pledged to plant 3 billion trees across member states by 2030 (1) by which time the United States will plant a further one billion trees (2), with Australia planning to match the US commitment by 2050 (3) while China is expected to plant some 35 million ha of new forest by that date (4). The magnitude of such efforts was underscored by the IUCN Restoration Barometer 2022 that reported that 14 million hectares of land are under restoration efforts globally and have already sequestered 145 million tonnes of carbon dioxide (5). In the United Kingdom, the government has pledged to plant 30,000 ha of trees annually by 2025 and maintain this rate until 2050, a historic elevation in the rates of afforestation (6) unseen since the 1970s (7–9). When executed in concert with decarbonization efforts across other emissions sectors, tree planting on this scale has the potential to play a central role in efforts to curb climate change (10, 11).

To move from policy pledges to growing trees, however, demands that a number of urgent decisions be made concerning how many trees to plant, of what species, and where, decisions that create trade-offs across carbon, agricultural, and timber production outcomes. The use of the Natural Capital approach for decision-making has been increasingly advocated in the academic and policymaking circles to resolve these trade-offs (12, 13). In essence, it imagines natural resources like trees as assets that provide services to mankind that can be captured and compared in monetary terms against the streams of income that could be generated by alternative uses of land, such as agricultural production. Applying the framework for net zero afforestation decisions thus means policymakers select the configuration of tree planting that delivers the best net benefits for society in the long run.

Unfortunately, those pressing choices must be made under conditions of significant uncertainty. As we show in this paper, the degree to which a program of afforestation and reforestation delivers carbon dioxide removal (CDR) services and how cost-effective those services are compared to other CDR technologies (14) depends critically on uncertain future environmental and economic conditions. Whether a particular tree species thrives or struggles when planted in a given location and the level of CDR services it can deliver depends on uncertain future environmental conditions (15). Similarly, the value society ascribes to those CDR services, the so-called Social Cost of Carbon (SCC), depends on uncertain future conditions (16, 17). In a high-emissions future, CDR services are highly valuable, but along a low-emissions trajectory, the benefits to society of drawing down atmospheric carbon are appreciably lower (17). Likewise, the financial costs of planting trees in a location are determined by the difference in the value of the marketed products generated by the forest (often principally timber) (18) and the products that could have been generated by that land in its alternative use (typically agriculture) (19–21). Again, the path those values will follow over time is highly uncertain being determined by yields and prices that reflect uncertain future environmental and economic conditions. In this paper, we consider how decision-makers should respond to this pervasive uncertainty and examine whether the risks associated with afforestation challenge the assumption that tree planting represents the most cost-effective CDR technology.

A tool commonly used by decision-makers to engage with uncertainty is scenario analysis. Scenario analysis involves the development of a set of logically consistent stories each describing a plausible future, and the assessment of how different policy decisions might play out under those particular futures (22). The latter information is usually derived with the help of models that relate land-use change to economic and environmental outcomes under each alternative assumption of future conditions (e.g. refs. 19, 23, and 24). Scenario analysis, however, has certain limitations. For one, those scenarios selected for analysis only represent some subset of possible futures. Decisions founded on that limited information space may prove wholly unsuitable under other possible future conditions. Likewise, as our research demonstrates, the best planting strategy under one scenario may prove to be among the worst under another. Scenario analysis fails to provide a coherent structure through which policymakers can resolve the inherent uncertainties that confront planting decisions.

In this paper, we pursue an alternative approach, reframing the problem as a portfolio investment decision and drawing on state-of-the-art, risk-averse optimization methods to identify planting strategies which are optimal given the range of potential futures. The portfolio analysis framework was initially developed for financial decision-making particularly for choosing the composition of investments in different risky assets like stocks, bonds, and commodities (25). Unlike scenario analysis, portfolio analysis examines the full range of predicted future conditions, specifying their joint statistical distribution. That information is then used to choose a portfolio of investments that strikes an efficient balance between risk and reward.

The portfolio approach has been shown to be broadly applicable to land use change problems like the forest planting problem we explore in this study. In this context, uncertainty about future climates and economic conditions complicates decisions on allocating land among different activities like conservation, forestry, and agriculture (26–30). While previous studies have examined the risks posed by either climate or economic variables in isolation, these risks are inherently interdependent and correlated. Their joint resolution ultimately shapes the outcome of land use decisions. Accordingly, this study examines the coevolution of economic risks and climate uncertainties and leverages the portfolio approach to identify forest planting strategies that take both sources of uncertainty into consideration. The planting strategy recommended by a portfolio analysis will tend to be one whose distribution of future returns does well on average while also limiting exposure to the possibility of very bad outcomes. Selecting such a planting strategy often involves seeking out what are termed “hedging” opportunities. Here, for example, trees of one species are planted in a given location because they tend to give good returns under future conditions in which trees planted in another location give poor returns, and vice versa. The planting strategy is a portfolio in the sense that it includes planting of both these varieties and thereby limits exposure to downside risk, the possibility of returns falling below a certain threshold. While previous studies have applied similar portfolio analysis frameworks to land use decision in order to reduce exposure to downside risks from climate change (31), this is one of the earliest times this framework been applied to analyze downside risks arising from coevolving climate and economic uncertainty.

This framework of analysis is globally applicable; however, we illustrate its use through a case study focused on UK tree planting in support of commitments to achieve Net Zero by 2050. Here, we characterize decision-makers’ uncertainty using the best current understanding of the joint distribution of future climatic and economic conditions and find that the natural capital value of tree planting (in monetary terms) varies significantly across the range of those conditions. Our findings reveal that planting strategies derived from portfolio optimization differ markedly from those designed to perform best under a single assumed future, as in traditional scenario analysis. Additionally, we explore the hedging possibilities afforded to policymakers through the strategic selection of different tree-species and planting locations. Finally, we compare the costs of meeting decarbonization targets from an optimal portfolio of tree planting to those of alternative CDR technologies based on bioenergy and direct air capture methods. Even when we adopt the extreme assumption that this alternative technology can deliver carbon capture without the risk of high costs to UK society, we find that it is outperformed by tree planting in all but extreme cases of highly risk-averse decision-makers or unrealistically low assumptions regarding the cost of the alternative technology.

## Results

Using a spatially explicit integrated environment-economy model of the United Kingdom (24), we estimated the net present value (NPV) of different tree-planting strategies over a planning horizon (2020 to 2050, discounted at 3.5% per annum) under 4,000 internally consistent realizations of future climate and economic variables—each referred to as a *climate*–*economy realization* (CER). Our NPV calculations capture three decision-critical components of the planting decision: the monetary value of the CDR services from planted trees; revenues from timber harvesting; and the costs of foregone agricultural profits from land on which trees are planted. The generation of CERs from an integrated model is critical in establishing coherent pathways for that set of variables. We find, for example, that the climate arising under different emissions futures not only drives particular patterns in the uncertain returns to woodland and agriculture but also shapes the SCC, that is to say, the value society attributes to carbon-removal services.

Following a typical scenario analysis approach, a standard approach applied to decision-making in the United Kingdom [such as the United Kingdom’s National Ecosystem Assessment (32, 33)], we initially focus on three CERs selected from across the range of possible futures confronted by policymakers in Great Britain. Those three scenarios comprise a near-historic emissions (NH) CER; a medium-emissions (ME) CER; and a high-emissions (HE) CER. These CERs are defined by the time paths of climate and economic variables illustrated in *SI Appendix*, Fig. S2. The economic variables modelled in the CERs encompass major drivers of the NPV of agriculture and forestry activities, including the prices of seven agricultural commodities (wheat, oilseed rape, barley, potatoes, sugarbeet, milk, beef, and sheep), fertilizer, timber and carbon prices. It also responds to climate variables (temperature and precipitation) representing a range of future emission scenarios [Representative Concentration Pathways (RCPs) 2.6, 4.5, 6.0, and 8.5]. As per UK policy aspirations, we assume that decision-makers wish to select a tree-planting strategy that delivers carbon sequestration totaling 12MtCO_2_e per year by 2050, a level consistent with attaining net zero at that point (34). A planting strategy consists of jointly choosing what species of tree (either broadleaf or conifer) to plant and in which location. At present, woodland in the United Kingdom comprises 49% broadleaf and 51% conifer species (35). Fast-growing conifers thrive in the cooler, wetter climates typical of the north, west, and southwest, delivering both carbon storage and commercial timber revenues. In contrast, slower-growing broadleaf species prefer warmer, drier areas as found in the southeast of the United Kingdom (Supporting Information *SI Appendix*, Fig. S4). Climate change projections show that many parts of the country will experience warmer and drier summers (36). Under moderate emission projections of future climate, the higher growth rates of conifers yield greater carbon storage than would broadleaf trees. However, under higher emission climates conifers suffer drought and depressed growth such that tree-planting strategies with a higher proportion of broadleaf trees sequester substantially more carbon by 2100 (37).

**Fig. 1.**
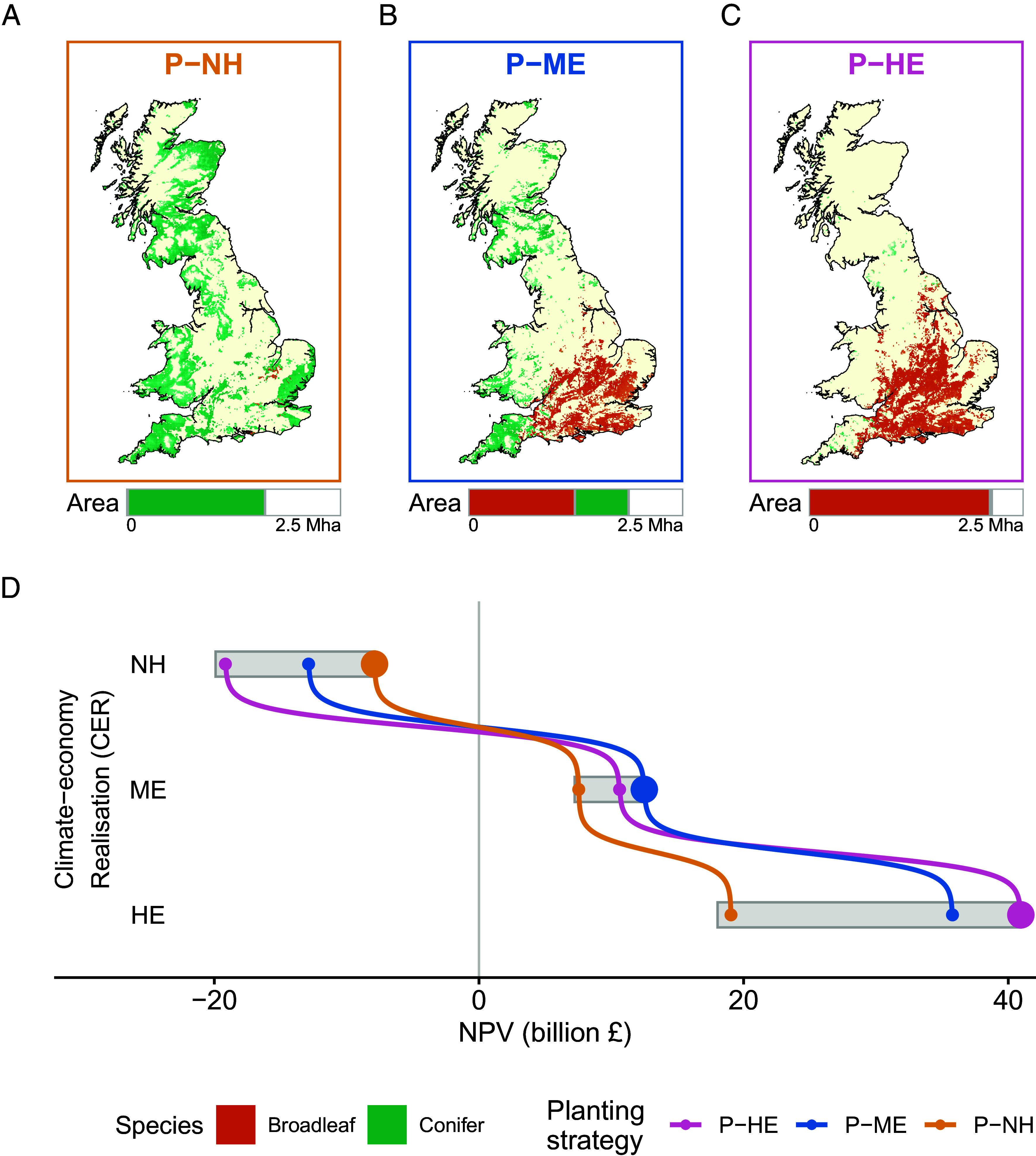
Natural capital outcomes of optimal woodland planting strategies under alternative CER each delivering the required 12MtCO2e annual sequestration target. (*A*–*C*) Shows the planting maps of three illustrative planting strategies with corresponding species mix (percentages show percentage of broadleaves): Planting under Near-Historic (P-NH); Planting under Moderate Emissions (P-ME); and Planting under High Emissions (P-HE), and the bar shows the area of planting by species (total bar width: 2.5 million hectares). (*D*) Shows the range of natural capital values obtained by 4,000 planting strategies (shaded rectangles) the NH, ME, and HE CER.

We use optimization tools to identify the planting strategy that delivers the maximum value under each scenario/CER. Those optimal planting strategies, labeled P-NH for the NH realization, P-ME for the ME realization, and P-HE for the HE realization, are plotted in the maps in Fig. 1 *A*–*C*.

Fig. 1 *A*–*C* clearly illustrates the fact that different future climate and economic conditions suggest drastically different optimal planting strategies to meet Net Zero commitments. Under NH conditions, the value delivered by planting conifers in less agriculturally intensive northern and southwestern parts of the United Kingdom consistently outweighs the possible benefits produced by broadleaves. As a result, the best planting strategy (P-NH) consists almost entirely of conifers (with only 1% broadleaf). The policy recommendation is modestly changed under the ME realization (66% broadleaf) and almost entirely reversed in the HE realization (99% broadleaf). Under HE conditions, the monetary benefits provided by broadleaf planting in agriculturally productive southern parts of the country dominate the benefits offered by coniferous planting in most parts of the country. Our analysis underscores the central problem that policymakers face when presented with information from a scenario analysis: Which tree-planting strategy should they adopt when the best strategy differs markedly from scenario to scenario?

The potential deficiencies of choosing to pursue the planting strategy recommended under the assumption of some particular scenario for future conditions are illustrated in Fig. 1*D*. We used our optimization tools to identify the NPV-maximizing planting strategy to meet carbon sequestration targets under each of our 4,000 CERs. We then evaluated the NPV delivered by each of those planting strategies under the NH, ME, and HE realizations. The range of resulting NPVs is shown by the shaded gray bars in the Figure. Observe that the NPV of all planting strategies tend to increase as we move from the NH to the ME to the HE realization, a phenomenon primarily reflecting the greater value society attaches to carbon sequestration under higher-emissions futures.

By definition, the strategies P-NH, P-ME, and P-HE are optimal under their respective CERs (e.g., the P-NH planting strategy is optimal under the NH realization). Of course, if we were to pursue some planting strategy alternative to P-NH when conditions turn out to be NH, then it would produce substantially inferior monetary value to UK society. Pursuing the P-ME planting strategy under NH conditions results in a planting strategy that is worth £12.4 billion less than the best planting strategy, whereas pursuing the P-HE planting strategy under NH conditions results in a planting strategy worth £19.2 billion less than the best strategy. Of course, while P-NH is optimal under NH conditions, it is suboptimal under ME; indeed, the optimal P-ME also satisfies the Net Zero carbon removal target while delivering £4.9 billion more in net benefits than does P-NH. Such differences are amplified when we move to the HE realization where climate change is more extreme and society places much greater value on carbon sequestration while the drier climate reduces farming profits in southern England, making broadleaf tree planting the preferred use of land in those locations (20, 23). Under this HE CER, pursuing P-HE instead of P-NH can lead to planting that delivers £21.9 billion more value to UK society.

The central message of this analysis is that a planting strategy designed to deliver optimally for one CER does not necessarily deliver well if the future follows a different climate and economic realization. For instance, while the P-HE strategy delivers significant net benefits under the HE realization, it is among the worst possible planting strategies under the NH realization. Likewise, while the P-NH strategy is optimal under low (NH) emissions, it misses valuable opportunities to deliver cost-effective carbon sequestration under high emissions (e.g., HE).

**Fig. 2.**
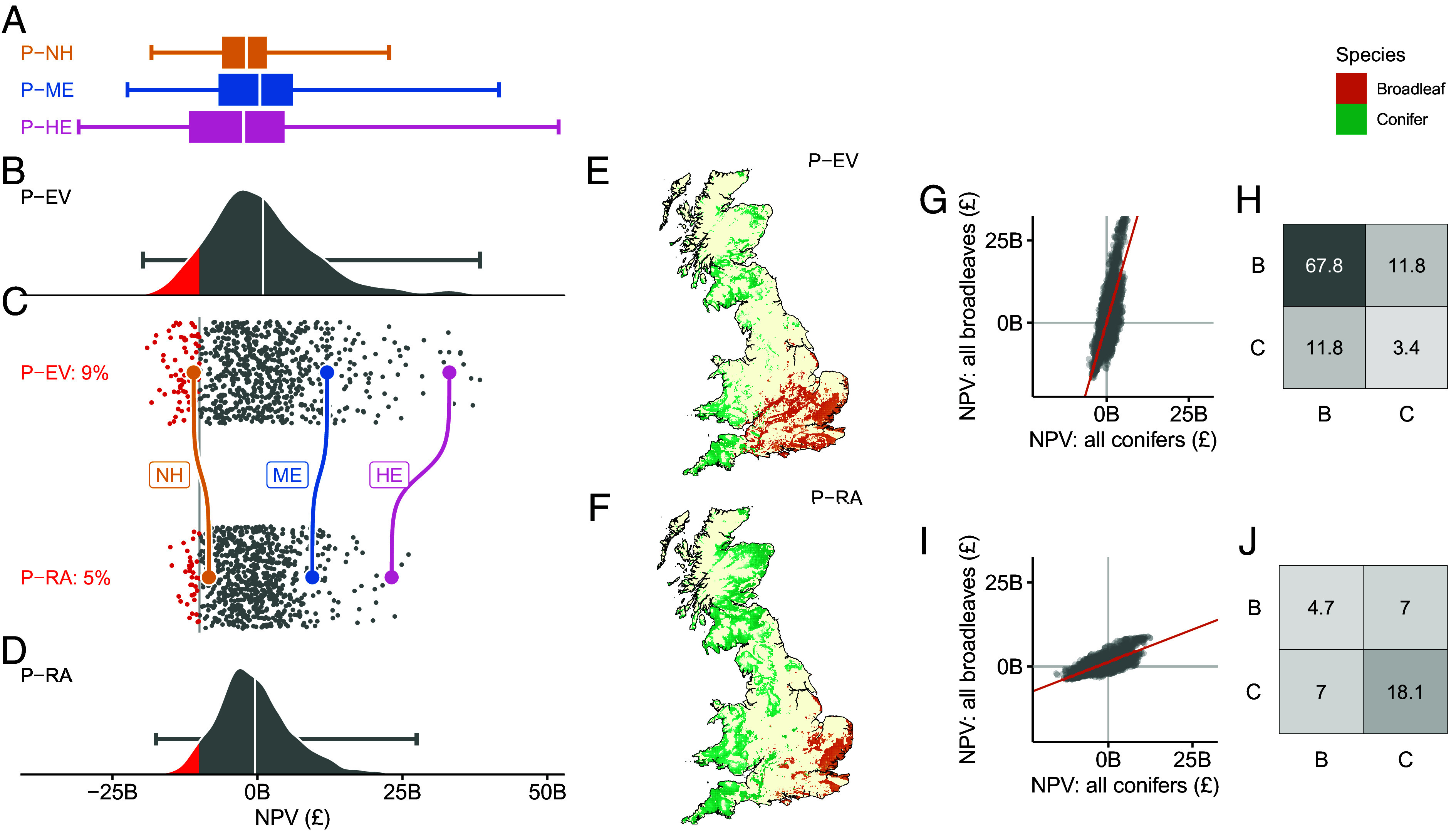
Risk-averse planting of diverse species reduces the chance of extreme losses. (*A*) Distribution of NPV from 4,000 CERs under P-NH, P-ME, and P-HE, showing minimum, 25th percentile, mean, 75th percentile, and maximum value. (*B*) Distribution of monetized net benefits for 4,000 CERs under the P-EV strategy with the horizontal line denoting the minimum and maximum of the distribution and the red shading denoting a user-defined risk threshold (losses exceeding £10 billion) which is replicated in the *Lower* panels of the Figure. (*C*) Shows a random subset of CERs and the corresponding net benefits achieved by the P-EV planting strategy (*Upper*) and P-RA planting strategy (*Lower*). CERs below the risk threshold (losses exceeding £10 billion) are again highlighted, and the probability of exceeding £10 billion losses shown. Outcomes of the P-EV and P-RA strategies under NH, ME, and HE are highlighted by the colored dots and lines joining the data points. (*D*) Distribution of monetized net benefits for 4,000 CERs under the P-RA strategy with the horizontal line denoting the minimum and maximum of the distribution; (*E* and *F*) spatial distribution of conifer and broadleaf planting across Great Britain under strategies P-EV and P-RA; (*G*) bivariate plot of the sum of NPV from all coniferous and all broadleaf planting in the P-EV strategy; (*H*) the variance–covariance matrix of the bivariate plot in *G*; (*I*) bivariate plot of the sum of NPV from all coniferous and all broadleaf planting in the P-RA strategy; and (*J*) the variance–covariance matrix of the bivariate plot in *I*.

Fig. 2 develops an alternative way of visualizing the outcomes associated with any given planting strategy, one that better aligns with the characterization of uncertainty used in portfolio analysis. Here, for each of our 4,000 CERs, we evaluate the costs and benefits associated with a particular planting strategy and describe our uncertainty over the returns that will be realized by that strategy as a probability distribution of NPVs.

Fig. 2*A* provides box and whisker presentations of the NPV distributions for planting strategies P-NH, P-ME, and P-HE. The wide distribution of NPV reveals the extent of risk underlying possible tree-planting strategies. None of these three tree-planting strategies are guaranteed to deliver positive net benefits—all three have some chance of generating negative NPVs. Furthermore, no single planting strategy results in a distribution of outcomes that is consistently superior to others across all CERs. The P-NH strategy is the least variable of the three (−£18.3 billion to +£22.7 billion), the P-ME planting strategy results in the highest overall expected value (+£0.4 billion), while the P-HE strategy has the highest best-case outcome (+£51.9 billion).

To discriminate between options, we need to understand the decision-makers’ preferences for risk. Here, we focus on two canonical risk preference positions: risk neutrality and risk aversion. A risk-neutral decision-maker would wish to pursue the planting strategy with a NPV distribution that has the greatest expected value (*Materials and Methods* and Eq. **13**). Following our labeling convention, we denote this the P-EV planting strategy. In contrast, a risk-averse decision-maker might wish to minimize downside risk exposure, a goal which in this study is identified as selecting the planting strategy with a NPV distribution that has the minimum conditional value-at-risk (CVaR) (*Materials and Methods* and Eq. **17**). We label this the P-RA planting strategy. We employ methods of optimization under uncertainty originally developed for portfolio investment problems in financial applications (25, 38) to identify the planting strategies that best deliver to those two differing objectives.

Fig. 2*B* details the NPV distribution arising from the P-EV planting strategy. By definition, this planting strategy produces a NPV distribution with the highest possible expected value, +£0.99B, a value which exceeds those resulting from the three planting strategies explored previously (P-NH: −£1.91B, P-ME: +£0.43B and P-HE: −£2.35B) or any other feasible tree-planting strategy. In focusing on expected value maximization, however, the P-EV planting strategy ignores other characteristics of the NPV distribution, including the range of possible outcomes. Nevertheless, Fig. 2*B* reveals that the range of outcomes under P-EV is considerably more condensed than that associated with P-HE (Fig. 2*A*), reducing worst-case NPV losses from −£30.8B to −£19.7B.

In contrast, the P-RA planting strategy is chosen to minimize downside risk. It achieves this by exploiting opportunities for risk diversification, choosing a portfolio of planting that ensures that under conditions where one species of tree planted in one set of locations delivers poor returns to society, some alternative set of trees is planted elsewhere which perform strongly under those conditions. More technically, it chooses a planting strategy with a combination of planting sites that have jointly low covariance. If, for illustrative purposes, we define “poor outcomes” as losses exceeding £10B (red-shaded areas in Fig. 2 *B*–*D*), the P-RA planting strategy almost halves the incidence of poor outcomes, reducing the probability of getting such an event from 9 (in P-EV) to 5%.

Yet, reducing risks comes with trade-offs. Choosing a portfolio of planting that minimizes downside risk is achieved in part by forsaking the opportunity to plant trees in locations that, under some possible futures, deliver very significant social benefits. Indeed, best-case outcomes under P-RA are significantly lower than those achievable under P-EV.

Fig. 2 *E* and *F* presents maps of planting locations chosen by the P-EV and P-RA planting strategies respectively. From those, it is evident that both strategies comprise a mix of broadleaves planted in the south and east with conifers planted in the north, west, and southwest. However, the P-EV strategy locates substantially more broadleaves in southern regions than P-RA, which instead places more emphasis on conifer planting in northern regions. This change in planting strategy suggests that the emphasis placed on conifers over broadleaves in the P-RA strategy is important in delivering risk reduction. Further light is shed on that supposition in Fig. 2 *G* and *I* which summarizes how the returns to broadleaf and conifer planting under the two strategies vary across the range of future conditions. Notice how under P-EV, widespread planting of broadleaves in the south leads to a very high dispersion of NPV outcomes for the broadleaf planting element of the planting strategy. The P-RA strategy achieves risk reduction by swapping some of the broadleaf planting locations most prone to delivering large downside outcomes, with coniferous planting. Dispersion in outcomes for broadleaf planting reduces substantially, though of course, there is an offsetting but relatively less substantial increase in the dispersion of NPV outcomes across potential futures for coniferous planting (Fig. 2*I*).

The P-RA planting strategy illustrates that the careful choice of species and location can reduce downside risk, though variability in the returns to tree planting remains high. One of the central difficulties in reducing risk in this context lies in the high degree of covariance in returns across candidate planting sites and species. That covariance arises through the existence of key uncertainties such as climate and agricultural prices that tend to have the same directional impact on returns for all tree species in all locations. In other words, when conditions result in planting being relatively costly for one tree species in one location, they also tend to be relatively costly for other species in other locations, limiting the possibilities for hedging risks. The importance of “global risk factors” in determining uncertainty over the benefits of portfolios of environmental interventions has been noted before in the context of risks to biodiversity conservation (39). In our tree planting problem, the phenomenon is illustrated in the covariance matrices in Fig. 2 *H* and *J*. These compare the returns arising from the broadleaf element of a planting strategy with that from the coniferous element across the range of possible future conditions. With the P-EV planting strategy (Fig. 2*H*), the returns from broadleaves exhibit very high variance (50.1) and there is relatively high covariance between returns to broadleaf and conifers (14.1). Risk-reducing planting choices under the P-RA strategy results in a reduced variance for broadleaf planting (4.6) coupled with an offsetting but less substantial increase in the variance of returns to coniferous planting (23.0). Moreover, P-RA exploits hedging possibilities, the success of which is reflected in the halving of the covariance in returns between the broadleaf and conifer elements of the planting strategy (covariance is 14.1 under P-EV and 7.4 under P-RA). Even so, covariance remains high, suggesting that hedging only serves to diversify some of the risk associated with adopting tree planting as a CDR technology.

Given the limits to risk reduction possible within a CDR strategy that relies exclusively on tree planting, consideration should be given to the possibility of incorporating other CDR technologies in the portfolio of assets delivering CDR services. If the returns to an alternative technology are uncorrelated with the global factors that drive returns to tree planting, then that alternative approach may have an important role to play in defraying risks in the cost of achieving decarbonization targets.

**Fig. 3.**
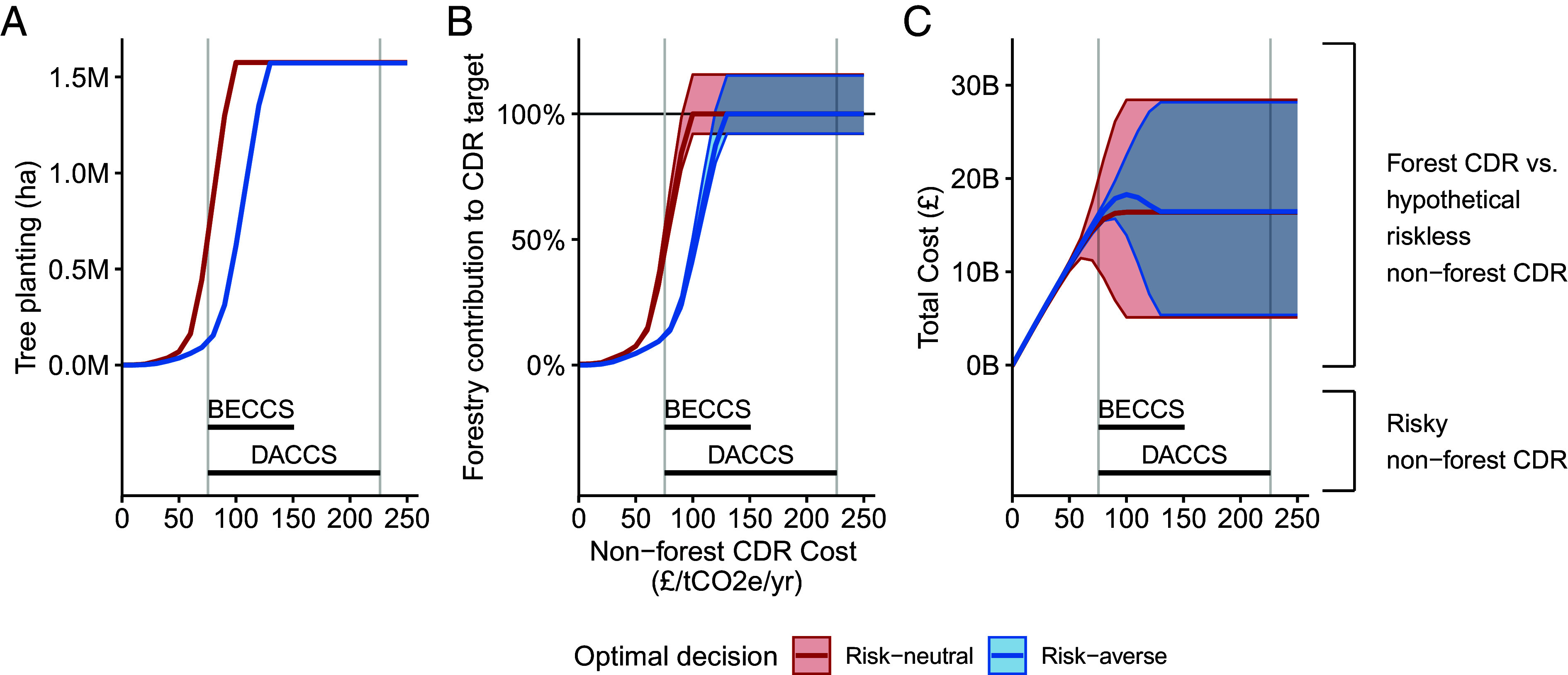
Tree planting remains a highly cost-effective approach to CDR compared to hypothetical risk-free alternatives. (*A*) The number of hectares of trees planted by a risk-neutral (red line) or risk-averse (blue line) decision-maker to meet the 12MtCO2e/year sequestration target as the per tonne cost CO2 removal (in £2020/tCO2e/year) of a hypothetical, risk-free, alternative CDR technology increases. (*B*) The contribution of that tree planting to the carbon sequestration target of 12MtCO2e/year. (*C*) Total costs of tree planting and the riskless CDR technology. Shaded areas show the full range of estimated carbon sequestration and costs from 4,000 modeled CERs. All plots also depict IPCC “medium confidence” cost estimates (in £2020/tCO2e) for CDR using Bioenergy with Carbon Capture and Storage (BECCS) and Direct Air Carbon Capture and Storage (DACCS) technologies.

Fig. 3 explores this mixed-technology portfolio strategy. Here, we introduce the possibility of deploying a hypothetical alternative CDR technology making the strongly optimistic assumptions that this can deliver up to the target sequestration rates of 12MtCO_2_e per year and that its cost risks are uncorrelated with the cost of tree planting. Now, our portfolio optimization algorithms not only select the locations and species for tree planting but also the optimal mix of this approach and deployment of the alternative CDR technology. As illustrated in Fig. 3, the relative mix of the tree and riskless CDR technologies depends on two factors: How expensive the riskless CDR is, and whether the decision-maker is risk-averse.

Consider first a risk-neutral decision-maker. Their objective is to select the mix of tree planting and the alternative CDR technology to achieve the target sequestration rate (12MtCO_2_e/year) so as to maximize expected value. Fig. 3 charts the outcome of such decisions across the range of possible costs associated with the riskless CDR technology, plotting the decisions of the risk-neutral decision-maker in red. Fig. 3*A* shows that if the riskless CDR technology is available at a cost of less than £50/tCO2e, then the decision-maker would elect to pursue that in favor of tree planting. At higher costs for the alternative CDR technology, trees begin entering the optimal portfolio of a risk-neutral decision-maker. At a cost of £75/tCO2e, that decision-maker would be best served planting 0.1 M hectares of trees, which deliver about 10% of the sequestration target as shown in Fig. 3*B*. The advantage of the riskless CDR technology over risky tree planting quickly diminishes as it becomes more costly. At £100/tCO2e, the optimal quantity of tree planting for the risk-neutral decision-maker increases to 1.5M hectares where 92 to 115% (11.0 to 13.9 MtCO_2_e/year) of the sequestration target will be met with tree planting (Fig. 3*C*).

A fully risk-averse decision-maker (blue lines) chooses the risk-free CDR technology over tree planting until the former reaches a cost of around £75/tCO2e after which they too add tree planting to their CDR portfolio. However, by the time the alternative technology costs £130/tCO2e or more, even a risk-averse decision-maker relies fully on tree planting to meet the 12MtCO_2_e decarbonization target. In this case, even though the alternative technology costs much more than tree-planting and both the risk-neutral and risk-averse decision-makers need to fully use tree-planting to reach the sequestration goal, the risk-averse decision-maker can still utilize a careful location and species to reduce a small amount of downside cost risks. When the alternative technology costs £250/tCO2e, the risk-averse decision-maker experiences £28.17B cost risk at the 95th percentile, which is £250M less than that experienced by the risk-neutral decision-maker at the same percentile.

Comparison with real-world (i.e., risky) alternative CDR technologies is provided below Fig. 3 *A*–*C* which provides medium confidence cost estimates for BECCS and DACCS as reported by the IPCC (14, 40). Even at the lowest levels of these costs both the risk-neutral and risk-averse decision-maker would be advised to include tree planting within their CDR portfolios.

## Discussion

Around the world, substantial and time-critical CDR investments must be made to avoid the worst impacts of climate change. For many countries, tree planting is mooted as an essential component of that investment. But tree planting is not a riskless CDR technology. Record-breaking heatwaves in Europe in 2003 and 2018 saw widespread tree deaths with stem dehydration disproportionately impacting conifer species (41, 42). In Sri Lanka, a substantial portion of newly planted trees do not survive and are affected by inappropriate planting sites (43). Tree disease and pest epidemics have seen a recent global surge and could undermine vital carbon sinks (44, 45). In California (United States), the future possibility of wildfires or sudden oak death has been cited as key risks to investments in trees as carbon offsets. Managing these risks through the careful development of risk-reducing planting strategies will substantially influence the extent to which tree planting contributes to decarbonization.

As illustrated by our research, such planting strategies can be identified through the application of investment portfolio approaches. The portfolio approach has been shown to effectively reduce risk in a number of other land-use investment settings (26, 28, 46–51). Our work extends its application in two ways: first in applying it to the problem of tree planting for decarbonization and second by examining uncertainties relating to both economic and climatic conditions. We show that the cost risks of carbon removal through tree planting can be mitigated through selecting combinations of planting sites and species that limit downside risk. Adopting this risk-averse approach to planting in the United Kingdom would limit the risk of experiencing extreme losses (defined here as greater than £10B) to just 5%.

We have demonstrated this approach to be successful for reducing risk associated with a portfolio of carbon removal activities and expect insights from this analysis to be applicable across a range of carbon removal targets. However, we cannot guarantee that meeting the carbon removal targets used to illustrate our approach will be sufficient to attain Net Zero at a national scale. For net zero commitments to be met, tree-planting targets must be continually adapted based on the future rates of carbon emissions reduction and sequestration across other emissions sectors.

Even with the adoption of risk-reducing planting portfolios, we find that the cost variability of tree planting in the United Kingdom remains relatively high. It proves difficult to hedge away the risks associated with tree planting because key drivers of uncertainty, particularly climate effects and the SCC, tend to impact the costs of planting similarly, irrespective of where planting takes place and which species is chosen in each location. The conundrum we presented in the United Kingdom is shared by several other nations where climate change is expected to adversely affect the forests critical to arresting climate change. Studies have shown the economic value of the current forest estate across the whole of Europe could be dramatically reduced under climate change (52), and a global study revealed that the negative impacts of climate change on forests’ natural capital could affect poorer countries much more significantly than wealthier ones (53). Countries must account for these predicted impacts on current and new forests when deciding where, what, and how trees are planted. Where countries can identify combinations of species and planting locations that jointly deliver low-correlation outcomes, particularly where a heterogeneous set of planting sites are available within a country’s jurisdiction, diversification across space and species can reduce risks by minimizing the chance of all tree species failing simultaneously in the same climate scenario (54). Nonetheless, our study shows that diversification alone is unlikely to fully mitigate the cost risks associated with planted forests assessed under a natural capital framework.

Of course, our analysis only allows for choice between two species, selected on account of being the dominant coniferous (Sitka spruce) and broadleaf (pedunculate oak) species in the United Kingdom’s national forests (8, 35). It is possible, that expanding the planting portfolio to include a range of species (perhaps even those not in the current UK forest estate), each potentially better adapted to some particular future conditions, may open up hedging opportunities increasing the resilience of delivery of CDR services from new forests under uncertain climatic and economic futures (30, 55). Moreover, future research utilizing the portfolio approach might identify further risk-mitigation potential by considering the complementary benefits and interactions of mixed-species plantations within the same site relative to the monoculture plantations considered here (56–58). Likewise, the adoption of forest management regimes that optimize for long-term carbon stocks rather than only mirror conventional practices as modeled in this study can further elevate carbon storage and manage associated risks (59, 60), benefitting from improved detail in models to account for differences in total planting costs driven by variations in management techniques and site accessibility. The portfolio approach demonstrated in our analysis provides the framework through which a decision-maker can be guided in choosing which, if any, of those tree species should be incorporated in that resilient planting portfolio.

The risk-diversifying portfolio strategy examined in this paper incorporates a range of CDR technologies beyond tree planting. As we show, even given the limited options for risk hedging through species and location choice considered, tree planting emerges as a low-regret option for CDR primarily on account of its relatively low costs. Indeed, tree planting forms part of the optimal CDR portfolio across the range of projected costs of alternative technologies such as BECCS and DACCS. Of course, the reason why those technologies exhibit such a wide potential cost range (Fig. 3) is because their implementation at the scales required has yet to be demonstrated (61–63). As such, decision-makers face real uncertainty over the potential costs of these alternative CDR technologies. Indeed, if one were to extend our work with an accurate characterization of the cost uncertainty in those alternative CDR technologies, it is likely to be the case that tree planting would dominate the CDR portfolio for a risk-averse decision-maker.

In such an analysis, a further reason to believe that tree planting will remain central to a resilient CDR investment strategy arises from possible correlation in the uncertain costs of tree planting, BECCS and DACCS. That correlation arises through land-use change. Like tree planting, BECCS makes large demands on land for the purposes of growing feedstock plants such as short rotation coppice willow or Miscanthus (63). DACCS, on the other hand, has a comparatively small land footprint but likely will require energy produced through expansion of renewable energy supplies that themselves need to be produced on land (64). Common demands on land-use resources suggest that when conditions lead to high (low) land costs, all technologies are relatively more (less) costly driving correlated cost risks across CDR technologies. As such, the additional deployment of these alternative technologies may not necessarily deliver very significant risk reductions, lessening the value of their contribution to a low-risk CDR portfolio.

One further reason to favor tree planting over other CDR technologies is that forests provide a wide array of ecosystem services in addition to CDR services. Such benefit flows include those from flood mitigation (65, 66), water quality enhancements (67), biodiversity gains for certain species (68), and improvements to noise and air pollution (69). Indeed, a wider analysis incorporating these effects into economic decision-making would likely change the optimal locations for tree planting in a way that delivers overall increases in social value (70, 71). Following the lessons of this paper, such analyses should acknowledge that the flows and values of these additional ecosystem services are also subject to uncertainties (12, 22).

While our analysis does much to support the strong presence of tree planting in CDR strategies, the prospect of technological change makes investment in alternative emerging CDR approaches worthy of consideration. Perhaps most importantly, early investments in those technologies could help resolve feasibility concerns and cost uncertainties (72). Decision-makers are effectively buying uncertainty-reducing information and providing themselves with the option to expand investment if those trials establish that alternative technologies are capable of delivering CDR at scale and at reasonable cost (73).

Even though our study characterizes many of the central uncertainties in the tree planting decision, numerous further uncertainties exist. While our analysis does not incorporate opportunity costs of tree-planting from potential urban land development revenues, we have extensively assessed the sensitivities of the planting strategies to future urban development scenarios and have revealed that excluding areas with potential future urban development has limited effect on the planting strategies’ NPV and spatial pattern (*SI Appendix*, Fig. S6 and Tables S2 and S3). Furthermore, our analysis does not consider the potential for technological change in agriculture, broad-scale shifts in global food production, consumption, and dietary preferences that might alter the opportunity costs of land used for tree planting. Likewise, uncertainties exist over other side effects of national tree planting through its endogenous macroeconomic price effects (74). “Leakage” effects also attenuate the CDR potential of national afforestation initiatives if planting displaces agricultural activities and increases emissions overseas (75, 76). Further, our study only models climate effects, not those arising from weather. Indeed, weather-related extreme events such as wildfires, heatwaves, and windfall have potentially severe impacts on forest CDR service flows (77–79), and their frequency and scale are highly uncertain in a changing climate (80, 81).

Notwithstanding the numerous potential avenues for expanding our analysis, our study demonstrates the effective integration of cutting-edge environment-economy modeling with modern methods of risk-averse optimization. This integration is able to provide detailed guidance to policy-makers when faced with complex environmental decisions confounded by pervasive climatic and economic uncertainties. In our UK case study, for example, we are able to identify tree-planting strategies that diversify risks and limit exposure to downside cost outcomes. Moreover, we are able to establish that, despite uncertainties over the net benefits arising from new forests, a carefully constructed portfolio of tree planting remains an indispensable component of a robust decarbonization strategy.

## Materials and Methods

### Study Area.

As part of its commitment to attaining net zero emissions of greenhouse gases by 2050, the UK Climate Change Committee has proposed a UK-wide target to establish 30,000 ha of trees a year by 2025 (9). Delivering on those targets would increase the country’s total woodland area from 13 to 17%, an expansion expected to offset emissions equivalent to 12MtCO_2_e per year by midcentury (34). A critical decision remains over which planting strategy to pursue, a decision which will define the set of locations where trees will be grown and hence the land that will be taken out of agricultural production.

The major consequences of alternative climate futures upon optimal tree-planting strategies sets up a challenge for decision-makers seeking to maximize the net benefits to society of tree planting. Within the United Kingdom (13) and increasingly globally (82, 83), official guidelines have adopted a natural capital approach (12, 84) to decision appraisal. This requires that all major benefits and costs, including those arising outside markets (such as the benefits of CDR), must be included within spending appraisals, typically by monetizing those values using an array of valuation tools (*ibid.*). This permits the use of standard economic assessment methods of investment appraisal such as the calculation of the NPV of a project. Here, we adopt such methods, seeking to maximize NPV over a typical 30-y time horizon (2020 to 2050) with the assumption that trees are planted in 2020, with net benefits discounted at a rate of 3.5% consistent with British policy-making recommendations (13).

While there are myriad costs and benefits associated with the planting decision (85, 86), for the purposes of this paper we focus on three components that capture the focal changes to natural capital value flows: the monetary value of carbon sequestration; revenues from timber harvesting; and the costs of foregone agricultural profits from land on which trees are planted, all of which are sensitive to future climate and economic variables (described in detail in *SI Appendix*). These values are assessed using the Natural Environment Valuation (NEV) suite of models (24, 86), a spatially explicit integrated environment-economy model of land use in Great Britain that have already been extensively used to inform national land-use decision-making (33, 71, 86, 87). NEV operates on a 2 km grid defining 57,230 gridded locations across Great Britain. Accordingly, we model the planting decision as selecting a set of cells across that grid and the species to be planted on the agricultural land in each chosen cell so as to optimize the relevant objective (e.g. maximizing expected value in the risk-free assessment) subject to satisfying the carbon storage requirement.

### CER.

The costs and benefits of a particular planting strategy are determined by a set of variables describing both future climate conditions and coevolving economic values including prices for timber and agricultural outputs as well as the SCC (88). When making the planting decision, however, the future pathway of those coevolving variables is not known with certainty. Our modeling framework captures that uncertainty by generating 4,000 different CERs, where each CER describes one internally consistent spatial and temporal pathway for those uncertain variables drawn from current empirical understanding of their possible distributions.

A key component of each CER is the assumed path of global emissions. Following standard practices in the climate sciences (89) we use realizations of climate conditions that conform with a set of four RCPs covering the broad range of predicted climate outcomes (90). We use the CHESS-SCAPE projections produced by the Centre for Ecology and Hydrology (91), which contains climate projections for four emissions pathways, and four members of the Regional Climate Model ensemble for each emissions pathway. Each CER has an emissions pathway and climate model member drawn independently with equal probability. Another key component of a CER is the assumed relationship between global temperature increases and loss in global economic productivity (also known as the “temperature-damage relationship”) which directly influences the SCC. We draw the temperature-damage relationship from a probability distribution modeled in ref. 92. A CER also contains other variables that depend on climate and the temperature-damage relationship, primarily the SCC, agricultural commodity prices (for the wheat, potatoes, rapeseed oil, sugarbeet, cattle, sheep, and milk production considered within NEV), and timber prices. A full description of how these variables were modeled from the climate and the temperature-damage relationship is given in *SI Appendix*.

Therefore, CERs differ widely in their predictions and outcomes even if they follow the same emissions pathway. For the purposes of illustrating key insights from our research, we selected three “focus” CERs: a “Near Historic” (NH) realization selected to typify the general features of the set of CERs drawn from RCP2.6; a “Moderate Emissions” (ME) realization selected to typify the general features of the set of CERs drawn from RCP4.5; and “High Emissions” (HE) realization selected to typify general features of CERs drawn from RCP8.5 (90), illustrated in *SI Appendix*, Fig. S2.

### Quantifying Uncertainties in Net Benefits from Tree Planting Activities.

We modeled changes in natural capital from the planting of a representative conifer (Sitka spruce) and broadleaf (pedunculate oak), in the arable land available in each grid cell. Cells where tree-planting activities lead to net emissions, for example through disturbance of soil organic carbon, were excluded.

Our analyses seek to identify which species of tree to plant and where to plant them in order to maximize the objective and achieve the carbon sequestration target *Q* (12MtCO_2_e per year). Since the rate at which trees sequester carbon differs from year to year as they grow over time, we take the yearly average carbon storage achieved over a single rotation period (where the rotation period is chosen to maximize timber revenues reflecting current common practice) to represent the annual sequestration services provided by growing trees. That quantity is calculated according to

[1] $$m_{\mathrm{ijs}}=\frac{1}{T_{\mathrm{ijs}}}\sum_{t=1}^{T_{\mathrm{ijs}}}g_{\mathrm{ijst}}\;\;\;\;\;\;\forall \;i,j,s,$$

where $m_{\mathrm{ijs}}$ is the average annual carbon storage delivered by planting species j in cell i under the climate pathway described by CER s. $T_{\mathrm{ijs}}$ is the rotation period for that tree species in that cell, and $g_{\mathrm{ijst}}$ is the marginal net storage of carbon in harvested wood products, deadwood, and soil in each year t as captured within the NEV system.

In achieving the sequestration target, choices of tree species and planting locations are made to maximize a monetary measure of social benefits. This measure encompasses net revenues from the planting and growing of trees for timber production (calculated using CER-specific timber prices), and the value of the carbon sequestered in trees (monetized using the CER-specific SCC). Additionally, it considers costs that comprise foregone profits from agricultural production on the land used to grow trees (calculated using CER-specific food prices).

In calculating the benefit flows that trees provide in timber and carbon sequestration, we again encounter the problem that their magnitudes differ markedly over time. The major cost of timber production, for example, is associated with the initial planting of trees, while the primary revenues arise only once those trees are harvested at the end of the rotation. As such, we choose to represent those uneven flows in the form of an equivalent annual benefit flow using the annualization of NPV approach calculated over a full rotation. The annualized benefit flows from timber are calculated as;

[2] $$r_{\mathrm{ijs}}^{\mathrm{Timber}}=\left(\sum_{t=1}^{T_{\mathrm{ijs}}}\frac{b_{\mathrm{ijst}}-c_{\mathrm{ijst}}}{{\left(1+\rho \right)}^{t}}\right)\frac{\rho }{1-{\left(1+\rho \right)}^{-T_{\mathrm{ijs}}}}\;\;\;\;\;\;\forall \;i,j,s,$$

where ρ is the discount rate, and $b_{\mathrm{ijst}}$ and $c_{\mathrm{ijst}}$ are, respectively, the revenues and costs from timber production in year t. Similarly, the annualized benefit flows of carbon sequestration is calculated as:

[3] $$r_{\mathrm{ijs}}^{{\mathrm{CO}}_{2}}=\left(\sum_{t=1}^{T_{\mathrm{ijs}}}\frac{g_{\mathrm{ijst}}{\text{SCC}}_{\mathrm{st}}}{{\left(1+\rho \right)}^{t}}\right)\frac{\rho }{1-{\left(1+\rho \right)}^{-T_{\mathrm{ijs}}}}\;\;\;\;\;\;\forall \;i,j,s,$$

where ${SCC}_{\mathrm{st}}$ is the social cost of carbon in year t along the SCC pathway dictated by CER s.

To simplify, we analyze the problem as if all planting occurs in the current period and that the decision-maker adopts a 30-year planning horizon. The value of planting trees of species j in cell i under CER s, therefore, is given by the NPV:

[4] $$R_{\mathrm{ijs}}=\sum_{t=1}^{30}\frac{r_{\mathrm{ijs}}^{\mathrm{Timber}}+r_{\mathrm{ijs}}^{CO_{2}}-r_{\mathrm{ijst}}^{\mathrm{Farm}}}{{\left(1+\rho \right)}^{t}}\;\;\;\;\;\;\forall \;i,j,s,$$

where $r_{\mathrm{ijst}}^{\mathrm{Farm}}$ are the farm profits from cell i in year t that are foregone on account of using that agricultural land to plant trees.

### Optimization Problem.

The policy-maker is confronted with the task of maximizing the value function, V, that quantifies the NPV of their CDR strategy:

[5] $$V\left(x,z|s\right)=\sum_{i=1}^{N}\sum_{j=1}^{J}R_{\mathrm{ijs}}x_{\mathrm{ij}}+z\sum_{t=1}^{30}\frac{{\text{SCC}}_{\mathrm{st}}-\gamma }{{\left(1+\rho \right)}^{t}}\;\;\;\;\;\;\forall \;s,$$

This function has two elements: a) the value of tree planting for CDR and b) the value of CDR from an alternative riskless technology. The tree-planting strategy is identified by the vector x, comprising elements $x_{\mathrm{ij}}$, which are binary decision variables identifying whether tree species j (from the J species in the analysis) is planted in cell i (from the N cells where planting could occur). Choices over deployment of the alternative CDR technology are given by z, a continuous variable identifying the quantity of that alternative technology (in units of MtCO2e/yr) to include in the solution. Deploying the alternative technology delivers CDR services valued using the SCC at a cost of γ per MtCO_2_e/yr which, because this technology is riskless, is constant across different CERs.

Choices over tree-planting strategy and deployment of the alternative CDR technology must satisfy a series of constraints as follows:

[6] $$\sum_{j=1}^{J}x_{\mathrm{ij}}\leq 1\;\;\;\;\;\;\forall \;i,$$

[7] $$\frac{1}{S}\sum_{i=1}^{N}\sum_{j=1}^{J}\sum_{s=1}^{S}m_{\mathrm{ijs}}x_{\mathrm{ij}}+z\geq Q,$$

[8] $$x_{\mathrm{ij}}\in \left[0,1\right]\;\;\;\;\;\;\forall \;i,j,$$

These constraints ensure that:

- a cell can only be used as a location to grow trees once and, in our analysis, those trees can only be of one species (Eq. **6**);
- the annual carbon sequestration target, Q, is met through the combination of the sequestration in planted trees, $m_{\mathrm{ijs}}$ (averaged across all CERs), and by the alternative CDR technology, z (Eq. **7**);
- the planting strategy variables are binary (Eq. **8**).

Since the optimization is constrained to deliver the target level of annual sequestration Q (Eq. **7**), no matter how that delivery is apportioned between sequestration in trees and sequestration by the alternative technology, the value of CDR services remains approximately constant across all solutions of x and z.

Therefore, the least-cost strategy to meet the target level of annual sequestration can be found by maximizing $V^{\prime }$:

[9] $$V^{\prime }\left(x,z|s\right)=\sum_{i=1}^{N}\sum_{j=1}^{J}\sum_{t=1}^{30}\left[\left(r_{\mathrm{ijs}}^{\mathrm{Timber}}-r_{\mathrm{ijst}}^{\mathrm{Farm}}\right)x_{\mathrm{ij}}-z\gamma \right],$$

The principal difficulty in defining the least cost CDR strategy, however, is that s is not known; that is to say, that the environmental and economic conditions that will be faced in reality are unknown. As such, V is stochastic from the perspective of the decision-maker.

### Scenario Analyses.

Our examination of a standard scenario analysis assumes that the carbon sequestration target is met by tree planting alone $\left(z=0\right)$. The problem is solved by assuming that some particular CER, $s^{\prime }$, is “true”, and integer programming methods are used to find the tree-planting strategy that maximizes V:

[10] $$x^{\prime }=\text{arg}\underset{x}{\text{max}}V\left(x,z=0|s=s^{\prime }\right),$$

Of course, the actual value delivered by a planting strategy depends not on the CER that is assumed by the decision-maker in choosing that strategy, but by the CER that is experienced in reality. To identify what that realized value might be, we take the tree-planting strategy x that is optimal under CER $s^{\prime }$, and then evaluate V under an alternative CER, $s^{\prime \prime }$:

[11] $$V\left(x=x^{\prime }\;,z=0|s=s^{\prime \prime }\right),$$

Fig. 1 *A*–*C* is constructed using this approach. Indeed, by repeating this calculation across the full range of possible CERs we are able to evaluate the full distribution of V for any given tree-planting strategy.

### Optimal Tree-Planting Strategies under Uncertainty.

We use linear programming to identify optimal planting strategies under any particular CER to meet the UK target of sequestering 12MtCO_2_e per year by 2050. In contrast to heuristic-based optimization approaches that identify several solutions of unknown optimality, linear programming can identify a single solution to the optimization problem with known optimality (93). Our initial analyses consider the decision problem where the 12MtCO_2_e p.a. target must be achieved through tree planting alone; subsequently, we explore how decisions might change if that carbon sequestration target can be met with a mix of tree planting and deployment of a hypothetical riskless CDR technology.

Given the function V that estimates the NPV of tree-planting strategy x under CER s, the problem of maximizing the value of tree planting to society under an assumed climate–economy realization $s^{\prime }$ can be written as

[12] $$\underset{x,z}{\text{max}}\;V\left(x,z|s=s\prime \right),$$

Of course, the “true” future climate–economy pathway is not known, such that from the point of view of a decision-maker, a tree-planting strategy is not characterized by one NPV but a distribution of NPVs defined across the range of possible CERs. Given that reality, a decision-maker might be better advised to choose a planting strategy whose distribution of outcomes under future possible realities has desirable properties.

One possible objective is to identify a planting strategy that maximizes the Expected Value (EV) of the distribution of outcomes. In that case, identifying the planting strategy that maximizes Expected Value (P-EV) amounts to solving the following problem:

[13] $$\underset{x,z}{\text{max}}\;EV\left(x,z\right)=\sum_{s=1}^{S}p\left(s\right)V\left(x,z|s\right),$$

where $p\left(s\right)$ is the probability CER s, which were constructed in such a way that we can assume each is equally likely to be a correct prediction of future conditions (i. e., $p\left(s\right)=\frac{1}{S}\forall s\in 1,\cdots ,S$).

Figs. 1 and 2 report findings where no alternative CDR technology is available, therefore z=0. In Fig. 3, we allow the alternative CDR technology to form part of the portfolio of CDR technology, and solutions in Fig. 3 are identified by iteratively solving for x and z for a range of values of γ.

Another possible objective is to choose a planting strategy that minimizes the chances of undesirable outcomes, which we refer to as risk averse (RA) decision-making. In this work, we quantify this downside risk using CVaR, also known as Expected Shortfall, a measure widely used in financial economics to quantify the risks associated with investment portfolios. The CVaR has been widely used as the standard for banks internationally as the recommended measure by the Basel Committee on Banking Supervision for quantifying risks and stress-testing in bank asset portfolios (94). The CVaR measure quantifies the average value of “poor outcomes” given that “poor outcomes” are defined as those that are smaller than a specified quantile of the distribution of outcomes. In contrast to typical land-use optimization applications that use variance as a measure of risk in land use (26–29), CVaR satisfies the properties of “coherent” risk measures found to be desirable in financial economics (95–98). CVaR thus remains a robust measure of downside risk even when the distribution of outcomes is nonsymmetrical (39, 99).

Shah and Ando (31) highlight the advantages of using a downside-risk measure (using a similar Lower Partial Moments approach) compared to a variance-based measure by testing the effectiveness of those at managing the effects of climate change uncertainty on the allocation of conservation resources. They found that, compared to variance-based approaches, downside-risk approaches are particularly suitable for reducing risks to climate outcomes that exhibit skewed distributions, and effectively diversify allocations compared to variance-based approaches. The authors also argued that even if outcomes are multivariate normal, variance still captures the wrong definition of “risk,” if the risk-averse decision-maker is only concerned about the possibility of returns falling below a certain threshold.

In this problem, we view realizations in V that are lower than a specified quantile in the distribution of V as “risky.” The objective is to maximize the value of the realizations that are deemed as “poor outcomes” so that the value of tree planting will still be relatively high in realizations that are worse than expected.

[14] $${\text{CVaR}}_{\beta }=-E_{s\in S}\left[V\left(x,z|s\right)\;|\;V\left(x,z|s\right)\leq q_{\beta }\right],$$

[15] $$q_{\beta }=\underset{\alpha \in \mathbb{R}}{\text{sup}}\left\{\alpha \;|\;\underset{s}{\text{Pr}}\left(V\left(x,z|s\right)\geq \alpha \right)\geq \beta \right\},$$

CVaR is the negative of the expected value of realizations in V that are lower than a quantile $q_{\beta }$, taken at the negative such that higher values of CVaR correspond to “riskier” outcomes. Here, β is a parameter taking a value between 0 and 1 that indicates the probability of the value V being larger than $q_{\beta }$. For this analysis, the parameter β is specified as 90%, implying that $q_{\beta }$ is the 0.1-quantile of the distribution of V and the undesirable outcomes are defined as the worst 10% of outcomes in the distribution.

As shown in Rockafellar and Uryasev (38), the tree-planting strategy that minimizes CVaR can be identified by minimizing the choice variable α, allowing us to express the CVaR of V in terms of x and z:

[16] $${\text{CVaR}}_{\beta }\left(x,z\right)=\underset{\alpha }{\text{min}}\;\alpha +\frac{1}{1-\beta }\sum_{s=1}^{S}p\left(s\right)\text{max}\left(-V\left(x,z|s\right)-\alpha ,0\right),$$

The optimal risk averse planting strategies, P-RA, is identified by minimizing this CVaR metric, or in other words, maximizing the negative of the CVaR.

[17] $$\underset{x,z}{\text{max}}\;\text{RA}\left(x,z\right)=-{\text{CVaR}}_{\beta }\left(x,z\right),$$

Like P-EV, P-RA is identified by setting z to 0 for Figs. 1 and 2 and solved for different values of γ to optimize the objective function in Fig. 3.

## Supplementary Material

Appendix 01 (PDF)

## Data Availability

Code used to produce analyses in this study is available at https://github.com/LEEP-Modelling-Team/tree-planting-uncertainty (100). The supporting data used to replicate results is available in Zenodo at https://dx.doi.org/10.5281/zenodo.14744237 (101).
