# Supplementary material for: Resilient tree-planting strategies for carbon dioxide removal under compounding climate and economic uncertainties
Source: Proc Natl Acad Sci U S A. 2025 Mar 3;122(10):e2320961122. doi: 10.1073/pnas.2320961122 (PMC11912443; doi:10.1073/pnas.2320961122)
Supplement: Supplementary file 1 — Appendix 01 (PDF) [file pnas.2320961122.sapp.pdf]

PNAS

Supporting Information for

Resilient tree-planting strategies for carbon dioxide removal under compounding climate and economic uncertainties

Frankie H. T. Cho * [a,b], Paolo Aglonucci [c], Ian J. Bateman [a], Christopher Lee [a], Andrew Lovett [d], Mattia C. Mancini [a], Chrysanthi Rapti [c] & Brett H. Day [a]

a. Land, Environment, Economics and Policy Institute, Department of Economics, University of Exeter, Exeter, EX4 4PU, United Kingdom
b. Centre for Biodiversity and Conservation Science and School of the Environment, University of Queensland, Brisbane, 4072, Australia
c. Institute for Sustainable Resources, University College London, London, WC1H 0NN, United Kingdom
d. School of Environmental Sciences, University of East Anglia, Norwich, NR4 7TJ, United Kingdom

Email: f.cho@exeter.ac.uk

This PDF file includes:

Supporting text
Figures S1 to S6
Tables S1 to S3

Supporting Information Text

Figure S1 gives a high-level overview of the methodology used to quantify the natural capital value of tree-planting in the study area for all climate-economy realisations (CER). The framework first models the distribution of future climate and economic variables as internally-consistent CERs. Here we first describe how the climate and economic variables are modelled. Based on these CERs, the Natural Environment Valuation (NEV) suite of models was used to quantify the natural capital values and carbon flux arising from possible tree-planting activities for each CER and each location that comprises of three focal changes to ecosystem services flows: (1) revenues from timber products, (2) monetary value of carbon sequestration, and (3) opportunity costs of agriculture.

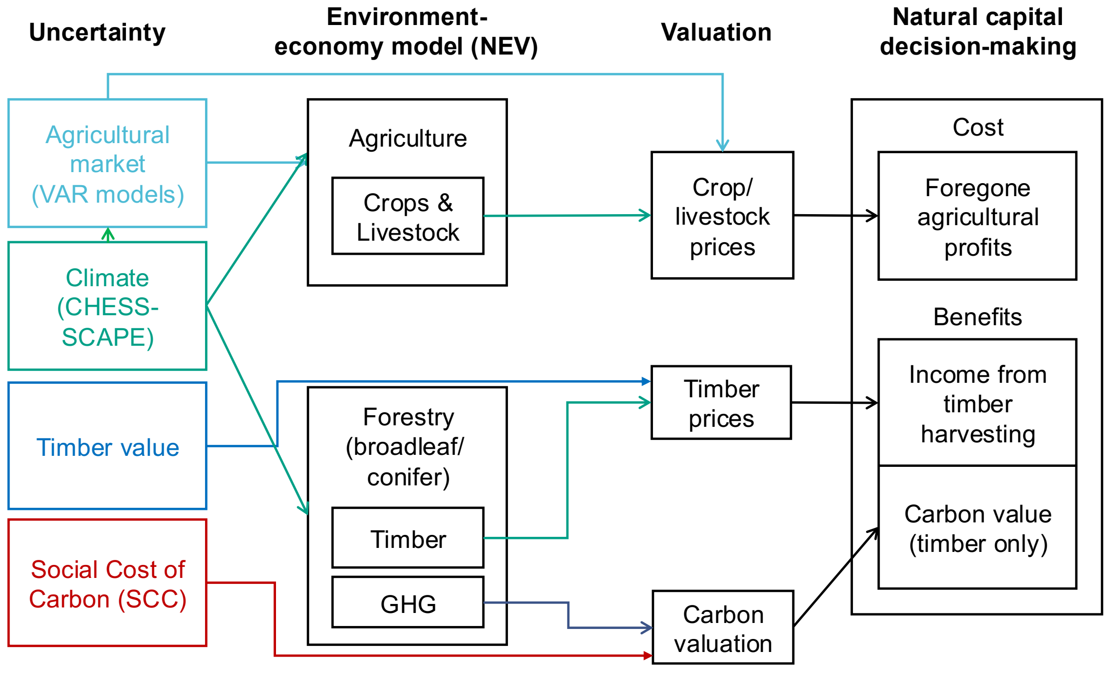

Fig. S1. A flowchart representing the methodology used to quantify uncertainties in natural capital values produced by tree-planting activities.

Uncertainty quantification

Climate variables. To quantify uncertainties over future climates, we use the latest high-resolution (1 km) climate projections from the CHESS-SCAPE dataset (1). The different projections in this dataset are taken to define the decision-maker's range of uncertainty over the future climate. The uncertainty captured in those projections includes both uncertainty over the possible future pathway for greenhouse gas concentrations in the atmosphere (described by RCP2.6, 4.5, 6.0 and 8.5) and uncertainty emanating from limitations in our ability to project future climates (characterised by variability across perturbed runs of a climate model under different RCPs). The dataset contained bias-corrected projections of climate variables at a 1km gridded resolution across the United Kingdom up to 2080 on a monthly basis. The climate projections are derived from downscaled projections from the UK Hadley Centre Regional Circulation Model (RCM) produced under the UKCP18 at the UK Met Office. The CHESS-SCAPE dataset extends the UKCP18 RCM projections by creating projections for RCPs other than RCP8.5. The dataset also provided projections of four climate model members for each RCP, indexed 01, 04, 06, and 15. These four climate members represent projections of the same RCM under different perturbed physics conditions. We extracted the values of future climate variables to match the 2km grid cell used in NEV. Subsequently, we calculated the growing season (April to September) temperature and precipitation projections of future climate variables on an annual basis up to 2080.

Future climates also drive the prices of goods and services produced by forestry and agriculture. The values of different agricultural produce are linked to future climates through macroeconomic vector-autoregressive models that capture long-term empirical relationships between climate, macroeconomic variables (e. g. GDP and interest rate) and the prices of agricultural commodities.

Value of agricultural commodities. Future climates also drive the prices of goods and services produced by forestry and agriculture. The values of different agricultural produce are linked to future climates through macroeconomic vector-autoregressive models that capture long-term empirical relationships between climate, macroeconomic variables (e. g. GDP and interest rate) and the prices of agricultural commodities. The Vector Autoregressive (VAR) models characterise the plausible distribution of prices of 7 major agricultural commodities: wheat, potatoes, rapeseed oil, sugarbeet, cattle, sheep, and milk. This output of VAR model is subsequently used to further characterise the trends of fertiliser and barley prices. To model possible co-movements of prices in agricultural markets, the prices of wheat, cattle sheep and milk are modelled simultaneously, whereas the prices of potatoes, rapeseed oil and sugarbeet are modelled independently. This leads to a set of 4 VAR models that can be used to predict the prices of the 7 agricultural commodities. These VAR models the historical variability and co-variability of prices of agricultural commodities.

Data on the trends in monthly Defra price indices (1990-2018) of these agricultural commodities relative to base year (2015) are modelled as a function of its own lag and other macroeconomic and weather variables. Macroeconomic variables include monthly data on Brent oil price ($/bbl), Real GDP (nominal GDP and GDP deflator) and monthly short-term interest rates. Weather variables include crop specific temperatures (measured in °C) and precipitation (measured in millimetres). Following established practice in the literature (2, 3), weather variables were constructed by averaging monthly weather observations based on a crop growing season (4) and the areas where the crop is cultivated (5).

All these models take the form of a system of equations, one for each variable, where each variable is modelled as a function of its own and other variables' lagged values.

$$\boldsymbol{Y}_t = \sum_{i=1}^{p} \boldsymbol{A}_i \boldsymbol{Y}_{t-i} + u_{it} \qquad (1)$$

$\mathbf{A}_i$ is a matrix of coefficients that describe the temporal lag of variables in the multivariate time series. $\mathbf{Y}$ is a k by 1 vector, p is the number of lags, and u_t are errors following this distribution: $\mathbf{u}_t \sim N(0, \Sigma_u)$. k is the number of variables in the multivariate time series.

In addition, responses of fertiliser prices (Defra agricultural price index) are modelled as a function of Brent oil price based on the Engle-Granger ADF Cointegration approach. $\mathrm{P_{fert}}$ is the Defra price index of fertilisers and $\mathrm{P_{oil}}$ is the price of Brent oil in $/bbl.

$$\ln\left(\mathrm{P}_{fert}\right)_{\mathrm{t}} = 2.015 + 0.583 \ln(P_{oil})_t \qquad (2)$$

Winter and summer barley prices in its Defra price indices are predicted with the exact same methodology as a bivariate relationship with wheat prices. $\mathrm{P_{barley}}$ is the Defra price index of barley. $\mathrm{P_{wheat}}$ is the Defra price index of wheat.

$$\mathrm{P_{barley,t}} = -0.103 + 1.015\ \mathrm{P_{wheat,t}} \qquad (3)$$

The 4 VAR models contain crop-specific temperatures and rainfall of wheat, potatoes, rapeseed oil and sugarbeet. To produce consistent predictions of future agricultural market predictions, predictions of crop-specific temperature and rainfall based on the percentiles of the spatially explicit climate time series were produced. For a given combination of percentiles of temperature and

rainfall, the temperature and rainfall data in the locations where the crops are planted based on predictions of the growing locations of wheat, potatoes rapeseed oil and sugarbeet in the United Kingdom obtained from NEV in 2020 were isolated. This allowed the construction of a time series of crop-specific temperatures specific to each percentile of temperature and rainfall specified within the climate time series. The temperature and rainfall data were subsequently averaged to produce one single time series for simulation in the VAR model.

Following the construction of these VAR models, a Monte Carlo approach was used to simulate random temporal trends from the year 2020 to 2050 conditional on the specified crop-specific temperature and rainfall time series (6). The VAR model provides a prediction of the most likely price trends of the agricultural commodities going into the future. Confidence intervals can also be estimated through the variance-covariance matrix of the errors: Σ_u. These intervals enable one to estimate the probability of a specific commodity reaching any certain price at any time step. Monte Carlo simulation of a VAR model allows one to simulate several plausible multivariate time series that reflects the temporal relationships between variables expressed in the temporal lag coefficients of the VAR model.

Social cost of carbon (SCC). Estimates of the temporal evolution of SCC are sensitive to both the future quantity of emissions and the estimates of the temperature-damage relationship that translate temperature increases to losses in global economic productivity (7). We characterise that uncertainty by drawing realisations of the temperature-damage relationship from a meta-regression model of a systematic review of these estimates (8). The uncertainty over temperature-damage relationship are propagated through a recently-developed Integrated Assessment Model (IAM) which updates the Dynamic Integrated Model of the Climate and the Economy (DICE) and reflects the latest findings in climate science and economics (9). Within a CER, therefore, we draw a temperature-damage relationship compatible with that realisation's assumed RCP. The time path for the SCC is then established by solving the IAM using that damage relationship and constraining the model to a pathway of global carbon emissions consistent with the assumed RCP as defined by the IIASA RCP database (10).

Our analysis considers two inputs that the SCC is sensitive towards: (1) the representative concentration pathway (RCP) and (2) the climate damage parameter, while noting that there are several other sources of uncertainties in the calculation of the SCC such as the equilibrium climate sensitivity to greenhouse gases, social discount rate and intergenerational inequality aversion parameters. We used the "Updated DICE" (Dynamic Integrated Climate-Economy model) with AMPL source code from Hänsel et al. (9) to estimate the SCC. Hänsel et al. updated Nordhaus' DICE model with modifications in the carbon cycle, energy balance model and updated climate change estimates. We made modifications on top of that to make the code sensitive to uncertainties over future emissions and temperature-damage relationship.

The updated DICE model, analogous to the original DICE model, seeks to find the optimal emission, temperature and carbon tax trajectories to maximise a utilitarian social welfare function discounted over time at a global level. The social welfare function is a function of the utility function U, the per capital consumption c, population L, and the discount factor on welfare $R(t) = (1 + \rho)^{-t}$:

$$\mathrm{W} = \sum_t U[c(t)]L(t)R(t) \tag{4}$$

The utility function has the form $U(c) = \frac{c^{1-\eta}}{1-\eta}$ where η is a parameter for generational inequality aversion. We used the social discount rate of $\rho = 1.5\%$ and an inequality aversion parameter $\eta = 1.45$.

Net output Q is a function of gross output Y, a Cobb-Douglas function of capital, labour and technology, minus damages Ω and mitigation costs Λ, defined as follows:

$$\mathrm{Q}(t) = \Omega(t)[1 - \Lambda(t)]\mathrm{Y}(\mathrm{t}) \quad (5)$$

Of particular interest is the damage function Ω. The damage function is defined as $\Omega(t) = D(t)/[1 + D(t)]$, where $D(t)$ is a function of global average temperature increases relative to historical averages T_{AT} and terms that describes the temperature-damage relationship ϕ_1 and ϕ_2 that translate temperature change to a percentage change in GDP.

$$D(t) = \phi_1 T_{AT}(t) + \phi_2[T_{AT}(t)]^2 \quad (6)$$

Published estimates of the temperature-damage relationship differ, suggesting vast uncertainties in the functional form of the damage function. While Hänsel et al. (9) set ϕ_2 to be fixed at the value at the "mean" of the preferred estimate reported by Howard and Sterner (8), we quantified the sensitivities in the SCC from the parameter ϕ_2 based on the probability distribution of their "preferred estimate", characterised by the mean and standard errors of the estimate, of Howard and Sterner (8). Each climate-economy realisation uses a different random draw of the estimate of the temperature-damage relationship. This allows us to characterise a range of possible SCC pathways for a given RCP.

Emissions are the sum of industrial emissions E_{Ind} and land-use emissions E_{Land}.

$$E(t) = E_{Ind}(t) + E_{Land}(t) \quad (7)$$

Where land-use emissions E_{Land} is specified exogenously and industrial emissions E_{Ind} is a function of carbon intensity σ and emissions reduction rate μ:

$$E_{Ind}(t) = \sigma(t)[1 - \mu(t)]Y(t) \quad (8)$$

We retrieved emissions data corresponding to each RCP from the IIASA RCP Database (10). We extend Hänsel et al. by using the corresponding land-use emissions scenario data from each RCP, as opposed to assuming exogenous land use emissions in RCP2.6. We further constrain the solution of the solver such that the emissions level match that in the RCP scenario.

The SCC is calculated as the change in economic welfare from an additional unit of CO_2-equivalent emissions, defined as a function of global emissions E and aggregate consumption C:

$$SCC = \frac{\partial W}{\partial E(t)} / \frac{\partial W}{\partial C(t)} = \frac{\partial C(t)}{\partial E(t)} \quad (9)$$

The SCC is used as the value of each tonne of carbon sequestered in each year.

Figure S2 depicts the predicted trends of climate and economic variables used in this study.

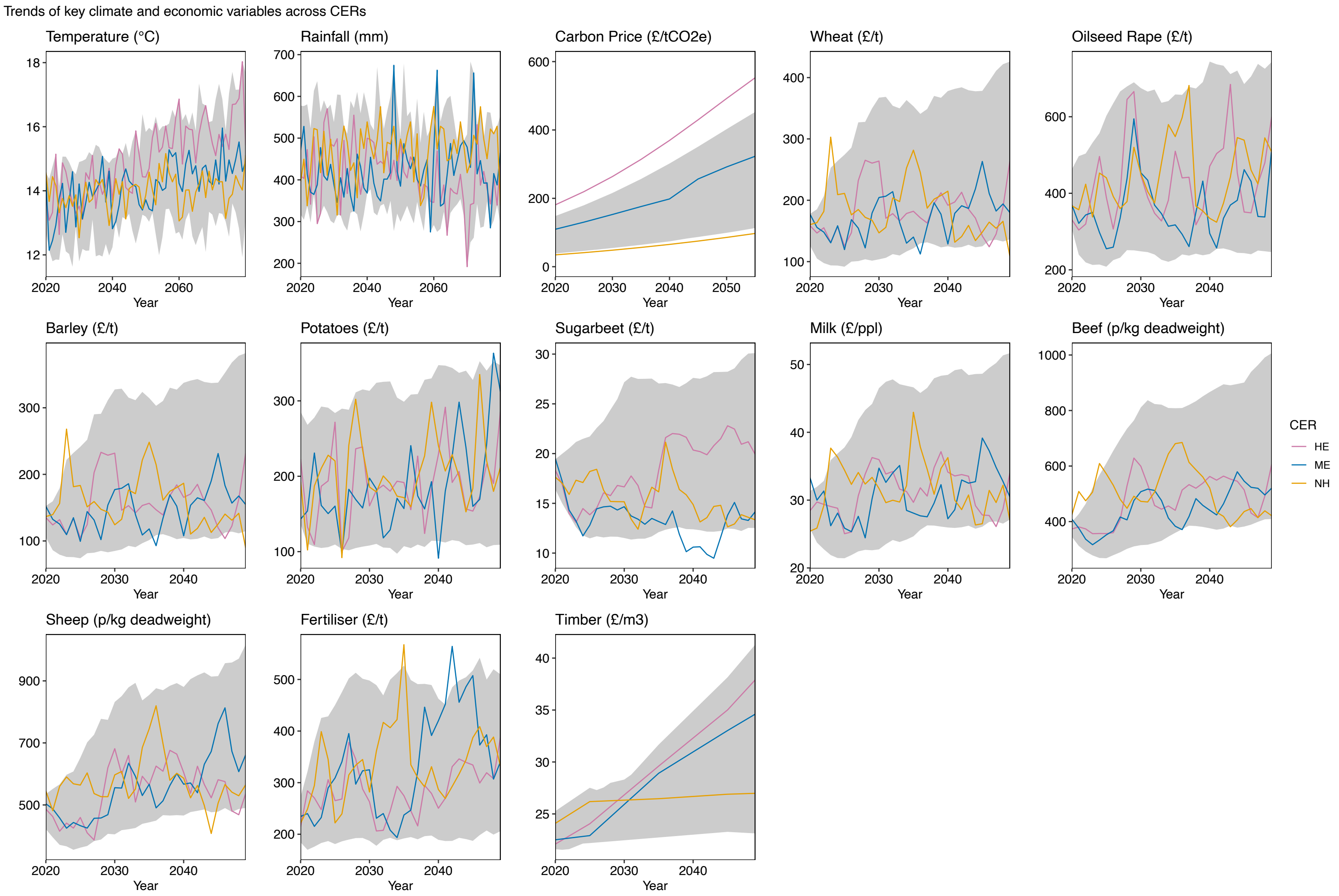

Fig. S2. Predicted trend in climate and economic variables across all the CERs, with the representative CERs highlighted. Shaded area represents 90% intervals across all CERs.

Valuation of changes in natural capital

The NEV suite of models is an integrated model of land-use in Great Britain that captures changes of multiple ecosystem services arising from land-use change developed at the Land, Environment Economics and Policy (LEEP) Institute at the University of Exeter. It predicts the change in the value of ecosystem services when land-use is altered through tree-planting. We built on top of the core functionalities of the forestry and agricultural models within NEV to quantify the uncertainties of these predictions under a wide range of climate and economic futures. The reader is referred to Day et al. (11) for a full technical description of the capabilities of the model inclusive of other ecosystem services, but here we present the description of the model workings relevant to this paper.

The model calculates the benefits and costs of tree planting. For each planted cell, NEV estimates the annualised quantity of timber output and carbon flux from the tree species grown in that cell under the realisation's assumed future climate. Those quantities are translated into monetised benefits using the realisation-specific future timber values and SCC time series. Emissions from agriculture displaced by tree planting activities are not expressly quantified because the carbon sequestration target (12MtCO_2e) identified as required to meet UK policy commitments does not account for avoided agricultural emissions. The costs of planting are the value of that stream of foregone outputs from farming, quantities being estimated by the NEV model's predictions of agricultural output choices driven by realisation-specific agricultural yields and prices. Finally, benefit and cost time series are discounted using the UK government social discount rate of 3.5% (12) and NPV is calculated over the standard 30-year time horizon.

Forestry. For this analysis, we predicted the change in natural capital value of land use of tree planting of representative tree species of conifers (Sitka Spruce) and broadleaf (Pedunculate Oak). Planting is restricted on arable and temporary grassland only and is assumed to completely displace agricultural activities on planted land. The net benefits of woodland planting are quantified as the difference between the natural capital benefits generated by tree planting, including timber revenues and carbon sequestration, minus the costs accrued from profits generated by agricultural production that are foregone because of tree planting. Incomes from timber harvesting are predicted with the aid of the CARBINE model (13) developed by Forest Research embedded within the NEV decision-support integrated environment-economy model. The model predicts forestry planting decisions and resulting timber output and profitability in response to characteristics, climate, and market conditions. It is assumed that a management regime exists over the newly created woodland on an annual basis between 2020 and 2060 for the species of trees planted. The model relies on the concept of yield classes in the framework of the Ecological Site Classification (ESC). The yield classes and site classifications are metrics describing the suitability of the piece of land for the growth of tree species. These metrics are functions of local and climatic factors. In the NEV model, the timber output of land is predicted through a two-stage process. First, timber volumes are predicted on an annual basis over the rotation period of the tree species with the CARBINE model. Second, the impacts of climate change on tree growth were predicted with a semi-parametric model. The semi-parametric model takes as inputs local and climatic factors including growing season temperatures/ precipitation, slope and elevation of the cell, geographical location, and soil characteristics (such as water regime, pH, water capacity and carbon in soil) to make predictions the future yield classes of the site.

Figure S3 illustrates the carbon storage outcomes of different woodland plantations under optimal management plans across different yield classes. While we assume that the location and species of tree-planting have to be determined at present, prior to when future climates are known to forest managers, and cannot be modified upon the start of the planting activity, the management practices (thinning and felling) adopted on the field can adapt to the climate variables that are experienced under particular CERs. Thus, management practices can continually evolve to respond to changing climates to maximise timber yield under each particular climate pathway.

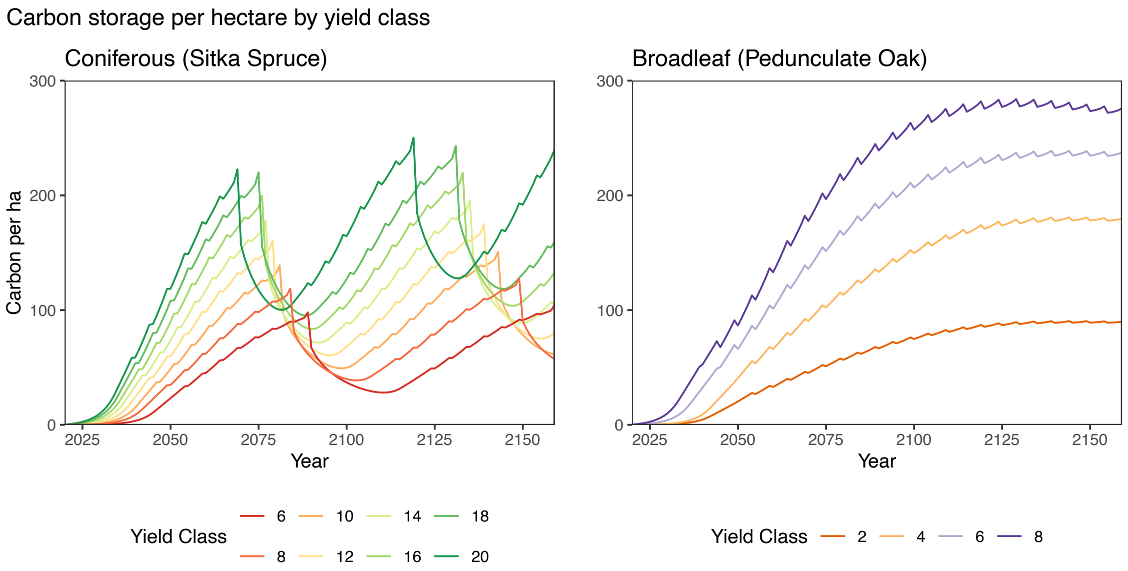

Fig S3. Aboveground Carbon storage per hectare across years across different yield classes.

The predicted yield classes of conifers and broadleaves strongly influences the amount of carbon storage possible for planting sites. Figure S4 illustrates the spatial variation in the predicted yield classes in the year 2080 across the country across all climate model members and emissions pathways, with carbon storage and yields generally being higher in higher yield classes. Overall, we observe that conifers have lower yield classes in high-emissions scenarios relative to low-emissions scenarios, the opposite is true for broadleaves, where yield classes are higher in high-emissions scenarios.

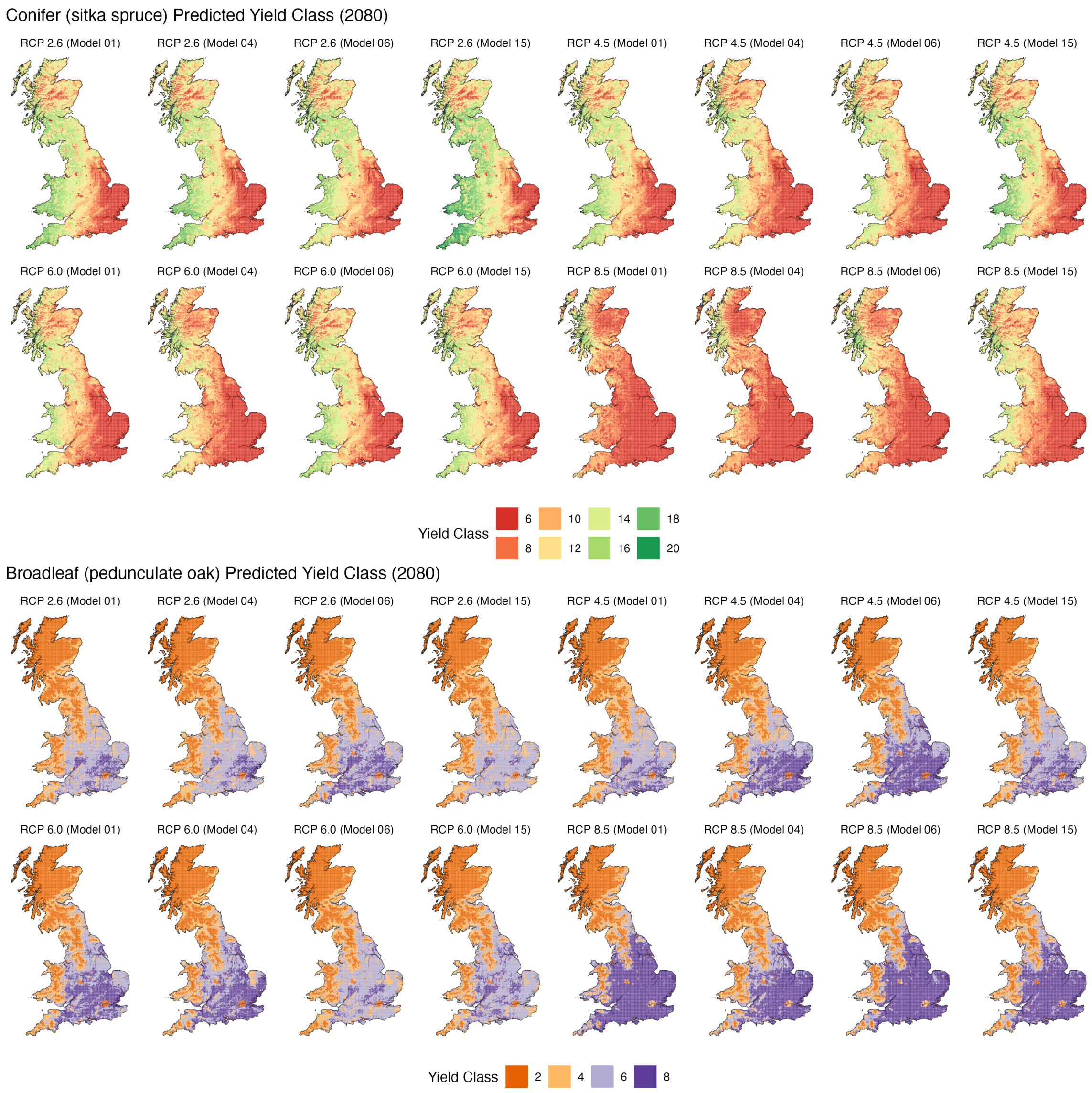

Fig. S4. Predicted yield class of conifers (Sitka spruce) and broadleaves (pedunculate oak) across all climate projections (represented by RCP and the number of the climate model), in the year 2080. Higher yield class (for the given species) suggests higher forest productivity.

We use the CARBINE model developed by Forestry Research to identify the management approach across all years that reflects the appropriate management practice for each climate pathway, based on the site-specific and year-specific yield classes predicted for each of the two species examined in this study. We computed the timber volumes predicted under each climate scenario, for each species across all sites to identify the yield and management costs for each tree species in each site. The timber volumes predicted through the CARBINE and the semi-parametric models are combined with the FC Forest Investment Appraisal Package to estimate the value of timber production and its associated management costs.

We utilised recently published results from a study of global forest sector models to model future time paths for the price of timber (14). The study modelled the global forest sector under 81 future pathway scenarios, including a range of RCPs (Representative Concentration Pathways) and the SSP (Shared Socioeconomic Pathways) storylines, and evaluates how these future pathways

affect outputs from three widely-applied models of the global forest sector, the Global Timber Model (GTM), the Global Biosphere and Management Model (GLOBIOM), and the Global Forest Product and Markets and Timber Supply Simulation model (GFPM). The authors produced a time-series of the predicted evolution of roundwood prices across each of the 81 future pathway scenarios.

We used all the 81 plausible pathways (3 models, 6 RCPs and 5 SSPs) from the outputs published in the paper and used the percentage change in the roundwood prices in the published output as the percentage change of timber prices in the CER. In each CER, the price path of the roundwood prices is set as a price path with the RCP of the CER, and the model and SSP randomly selected within the set of price paths with that RCP, using the following equation:

$$P_{timber,t}^{s} = P_{timber,baseline} \times \frac{I_{timber,t}^{s}}{I_{timber,2015}^{s}}$$

Where the price of timber in the UK at time t for CER s is $P_{timber,t}^{s}$, the price of timber in the UK at 2015 is $P_{timber,baseline}$, $I_{timber,t}^{s}$ is the global price of roundwood modelled in the study for the climate-economy realisation s at time t, and $I_{timber,2015}$ is the baseline price (of 2015) used in the study. The timber price is then reflected in the revenues from timber scales.

By estimating the difference between revenues from timber sales and costs from management, profits are calculated in a Net Present Value terms using the same social discount rate of 3.5%. These Net Present Values are then annualised over one rotation period and constitute the timber revenues component of the tree planting alternatives. Details of this methodology can be found in Binner et al. (15). The natural capital value produced by carbon sequestration is estimated by multiplying the change in carbon volume for any given year with the Social Cost of Carbon (SCC) in that year, described in the previous section. The same discount rate (3.5%) was used to convert earnings from one rotation to Net Present Values. These earnings are subsequently annualised by dividing the total NPV with the number of years in the rotation period.

Foregone agricultural profits. The costs from foregone agriculture were estimated with a farm management model building on several years of spatial econometric modelling of British agriculture (16–18). Earlier iterations of the model formed a crucial component of the UK National Ecosystem Assessment (18). The agriculture model predicts farm planting and stocking decisions in response to land characteristics, climate, and market conditions. The model is estimated primarily using data from the June Agricultural Census (1976-present, Defra), and the Farm Business Survey (2007-2015, Defra). Each location is taken as an agricultural decision unit managed by a "farmer." The model assumes that a "farmer" manages the agricultural activity in each location. Crucially the "farmer" decides land use for the current year based on prices of agricultural commodities and weather observed in the previous year. The following process is repeated for each price/ climate scenario, across all locations and in each year:

1. At year t, a biophysical model predicts the share of arable and grazing land in that year based on soil and other site characteristics as well as growing season temperatures and precipitation of that particular year. Details of the biophysical model can be found in Ritchie et al. (19).
2. Given the allocation of arable and grazing, the "farmer" observes the prices of agricultural commodities, fertiliser, and growing season temperatures/ precipitation of the year. These prices are obtained from the multivariate time series simulated in the climate-economy realisation. The "farmer" decides what crops to plant/ livestock to raise for t+1 based on the observed prices of agricultural commodities and biophysical conditions of the farm.
3. At the year t+1, the "farmer's" profits are calculated based on the land use allocation decided during t and the realisation of prices and climate in that year
4. The opportunity cost of planting in that year is calculated as the agricultural profits in the arable land and temporary grassland portions of the land. If tree planting occurred those

pieces of land, it will not be agriculturally productive and therefore these profits will be the opportunity cost of planting

5. The algorithm is repeated for the next year (t = t+1)

After the algorithm estimates the spatial opportunity costs of planting in the years 2020 to 2050, the costs are annualised with a discount rate of 3.5% for all locations.

Summary statistics

Table S1 depicts the summary statistics of the planting strategies (P-NH, P-ME, P-HE, P-EV, and P-RA) evaluated across several metrics: (1) its NPV distribution, in terms of its mean, NPV under the CERs NH, ME and HE, minimum, maximum, CVaR and probability of losses greater than £10 billion, (2) Planted Area of conifers and broadleafs, (3) expected carbon sequestration across tree species, and (4) the carbon sequestration under the different representative CERs.

	P-NH	P-ME	P-HE	P-EV	P-RA
**NPV distribution (billion £)**					
Mean	-1.912	0.428	-2.353	0.991	-0.411
NH	-7.892	-12.876	-19.162	-11.002	-8.382
ME	7.538	12.492	10.627	12.012	9.47
HE	19.045	35.781	40.947	33.104	23.141
Minimum	-18.277	-22.405	-30.848	-19.711	-17.495
Maximum	22.702	41.69	51.938	38.373	27.435
CVaR-0.9 (higher the better)	-11.114	-14.346	-21.032	-12.36	-10.392
Probability of losses greater than £10 billion (lower the better)	0.067	0.13	0.303	0.09	0.05
**Planted Area (million ha)**					
Conifers	1.597	0.625	0.021	0.673	1.261
Broadleaf	0.017	1.24	2.108	1.053	0.315
Total	1.615	1.865	2.129	1.726	1.576
**Expected MtCO_2e/year**					
Conifers	11.844	4.421	0.215	4.875	9.129
Broadleaf	0.156	7.579	11.785	7.125	2.871
Total	12	12	12	12	12
**MtCO_2e/year by CERs**					
NH	11.5	11.4	11.4	11.5	11.5
ME	12.0	11.7	11.9	12.0	12.0
HE	11.7	11.7	11.6	11.5	11.6

Table S1. Net Present Value (billion £), Planted Area (million ha), Expected annualised MtCO2e (averaged across all CERs), and annualised MtCO2e of each focus CER, of the planting strategies P-NH, P-ME, P-HE, P-EV and P-RA.

Sensitivity analysis

We conducted a range of sensitivity analyses to enhance the comparability of our results with other analyses and address potential shortcomings of the modelling approach. Here, we detail the approach used to examine the sensitivity of our findings across alternative model specifications.

Planting strategies under specific RCPs. First, we examined the set of optimal planting strategies that assume the future emissions pathway (RCP) is known for certain. Such an analysis will provide insights to practitioners identifying the optimal set of planting strategies in a potential future where the possibility of some RCPs is ruled out to be impossible. Note that resolving the future emissions pathway (RCP) does not fully resolve the set of uncertainties modelled in this paper, as there are many CERs (1,000 per RCP in this analysis, including four distinct future climate projections per RCP) that represent the range of possible futures even if the world is committed

towards one particular RCP. Therefore, the analytical tools applied in this paper are still useful even if future emissions pathways are known.

To conduct this analysis, we assigned the probability of the CERs that are not from a specific RCP to be 0, and each CER within the RCP to be $1/N_{CER}$, where N_{CER} is the total number of CERs consistent with the RCP. This analysis resulted in four distinct planting maps, each representing the optimal planting strategies under a particular assumed future emissions pathway. We repeated our analysis with both the EV and RA algorithms to identify the optimal planting strategies using these differing approaches. All these planting strategies, including the spatial location, mix of species and NPV distribution, are represented in Fig S5.

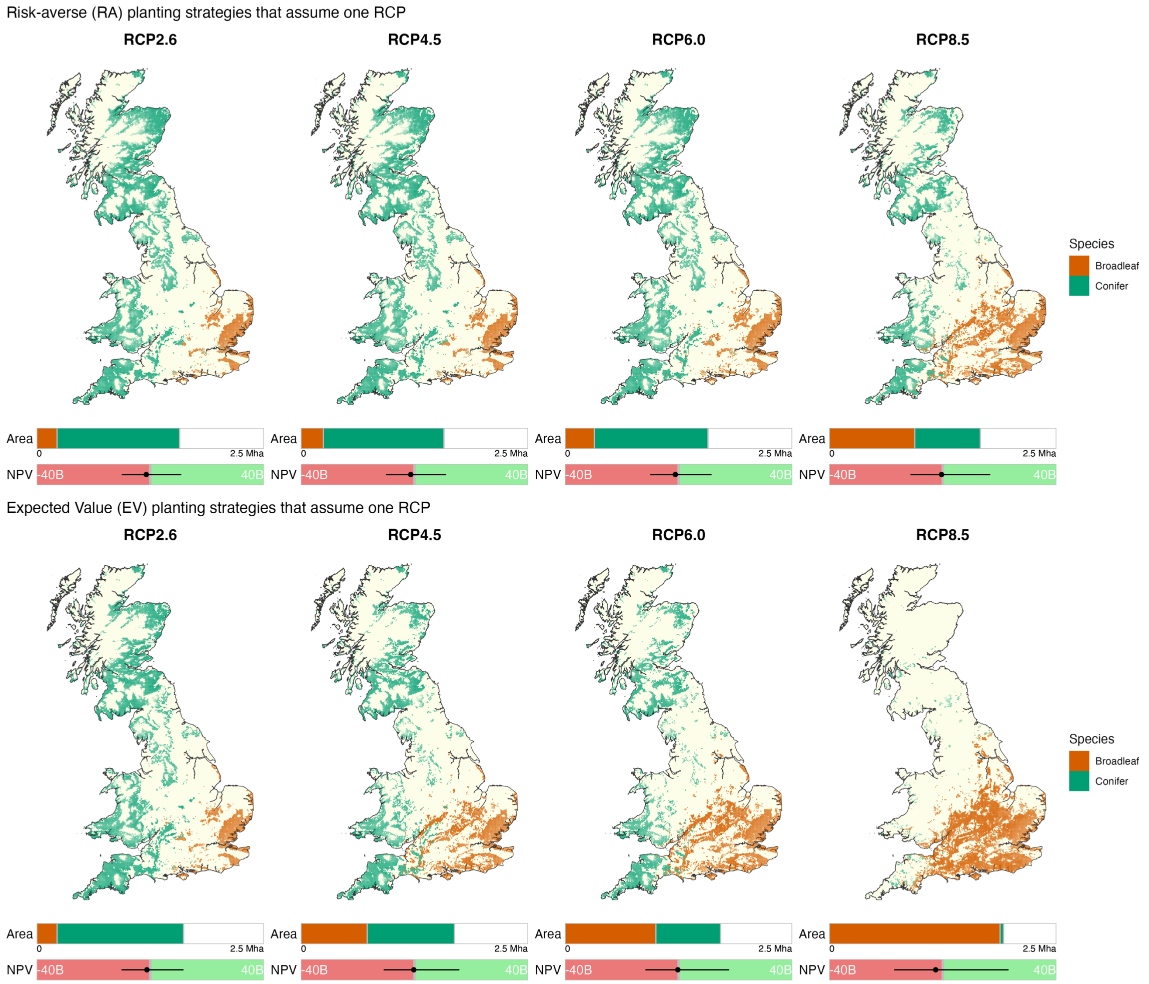

Fig. S5. Planting strategies where a particular RCP is assumed to be "true" and other RCPs are assumed to be improbable (i.e., probability equals to zero), identified across the different RCPs and different strategies (Expected Value and Risk-averse), with bars indicating planting area by species (bar width: 2.5 million ha) and NPV.

As we see, the assumed emissions pathway has a strong impact on the planting strategies, including the location and mix of species planted. Overall, the mix of broadleaves relative to conifers increases in higher emissions pathways. Crucially, this analysis also revealed that the method to which the planting strategy was identified still have a strong impact on the location and mix of species, even under the same assumed RCP. For instance, we observe that the P-EV planting strategy would plant 98% as broadleaves under RCP8.5, whereas the more risk-averse P-RA would only plant 57%.

Accounting for the potential for urban development. A limitation of the modelling framework is that it only models the opportunity cost of agricultural activities from woodland planting and not of alternative uses of land, such as urban and housing development. A first-best approach to account for this shortcoming, and something that was technically feasible, was to incorporate a model of future urban/ housing development revenues into the optimization framework. Such a modelling framework would more accurately reflect the opportunity costs of forestry; in areas with high development potential, the opportunity costs of woodland planting would be much higher than foregone agricultural profits and would rather be more accurately modelled as the foregone profits from housing development. However, to our knowledge, a spatially explicit economic model of future urban/ housing development revenue that is sensitive to future climate and macroeconomic variables is yet to exist.

Indeed, urban development can exert significant influences on the optimal tree-planting strategy and resultant NPV of these activities. If planting were occurring in lands with high future value of urban land, the opportunity costs of these planting activities will be much higher than what was reported. This means that tree-planting would optimally take place in areas that are not likely to be developed into urban areas (due to low future development potential).

To evaluate whether accounting for future urban development value will likely exert significant influences on the modelling results, we make use of recently released outputs from the CRAFTY-GB model (20). The CRAFTY-GB model is a large-scale agent-based model that uses a range of land system data to project future urban development in Great Britain at decadal timesteps and 1km^2 resolution, across several SSP and RCPs pairings. While not providing predictions of the value realized from land by urban development, CRAFTY-GB provides some of the only estimates of future urban development in GB currently available. We obtained the land-use change projections from the model and extracted the areas in which urban development is projected to occur by the year 2050, the end of the planning horizon in our analysis.

We conducted two analyses to assess the impact of future urban development on our model. In our first set of analyses, we tested the proportion of planting identified in the optimal planting strategies in the main analysis that overlapped with areas of projected future urban development. This is to assess the degree to which our optimal planting strategies are likely to change if we instead used a model that accurately captured the opportunity costs of planting from precluding future urban development. The percent of planting (in terms of area) that overlapped with future urban areas are depicted in Table S2.

	2050 Urban Land-use	P-NH	P-ME	P-HE	P-EV	P-RA
**RCP2.6-SSP1**	No	1575725 ha (98%)	1800450 ha (97%)	2031190 ha (95%)	1671164 ha (97%)	1537034 ha (98%)
**RCP2.6-SSP1**	Yes	38902 ha (2%)	64591 ha (3%)	97490 ha (5%)	54967 ha (3%)	38778 ha (2%)
**RCP4.5-SSP2**	No	1577708 ha (98%)	1801074 ha (97%)	2027232 ha (95%)	1671977 ha (97%)	1538466 ha (98%)
**RCP4.5-SSP2**	Yes	36919 ha (2%)	63966 ha (3%)	101448 ha (5%)	54154 ha (3%)	37346 ha (2%)
**RCP4.5-SSP4**	No	1578792 ha (98%)	1803418 ha (97%)	2035146 ha (96%)	1675413 ha (97%)	1539145 ha (98%)
**RCP4.5-SSP4**	Yes	35835 ha (2%)	61623 ha (3%)	93534 ha (4%)	50717 ha (3%)	36667 ha (2%)
**RCP6.0-SSP3**	No	1572331 ha (97%)	1794586 ha (96%)	2023213 ha (95%)	1665571 ha (96%)	1533780 ha (97%)
**RCP6.0-SSP3**	Yes	42295 ha (3%)	70454 ha (4%)	105467 ha (5%)	60560 ha (4%)	42032 ha (3%)
**RCP8.5-SSP2**	No	1577708 ha (98%)	1801074 ha (97%)	2027232 ha (95%)	1671977 ha (97%)	1538466 ha (98%)
**RCP8.5-SSP2**	Yes	36919 ha (2%)	63966 ha (3%)	101448 ha (5%)	54154 ha (3%)	37346 ha (2%)
**RCP8.5-SSP5**	No	1541358 ha (95%)	1745017 ha (94%)	1956776 ha (92%)	1619701 ha (94%)	1502604 ha (95%)
**RCP8.5-SSP5**	Yes	73269 ha (5%)	120023 ha (6%)	171904 ha (8%)	106429 ha (6%)	73208 ha (5%)

Table S2. Percent of overlap of planting strategies in the main analysis with the areas of projected future urban land-use across RCP-SSP pairs predicted by the CRAFTY-GB model

Here, we observe that the percent of overlap with future urban areas (relative to the percent that did not overlap) varies depending on the planting strategy (in the columns) and the future land-use projections adopted. An overarching pattern we see though is that the percent of planting in the planting strategy that occurs in projected future urban areas, irrespective of the land-use projection or planting strategy, is always below 10% of the total planting in the planting strategy. In most planting strategies and land-use projections, less than 5% of planting occurs on projected future urban land, and in the most extreme case, only a 8% overlap occurs (RCP8.5-SSP5 under P-HE), likely driven by the fact that P-HE has a high percent of broadleaved planting that generally fares better in the southern, more urbanized, part of Great Britain, and the fact that more urban development is projected to occur under SSP5 (Fossil-fueled development).

In our second analysis, we excluded land where urban development is projected to occur across the different RCP and SSP projections and reran our algorithm to identify the optimal planting strategies under the same set of CERs consistent with the RCP and calculated the net present value of the planting strategy. These results are presented in Figure S6.

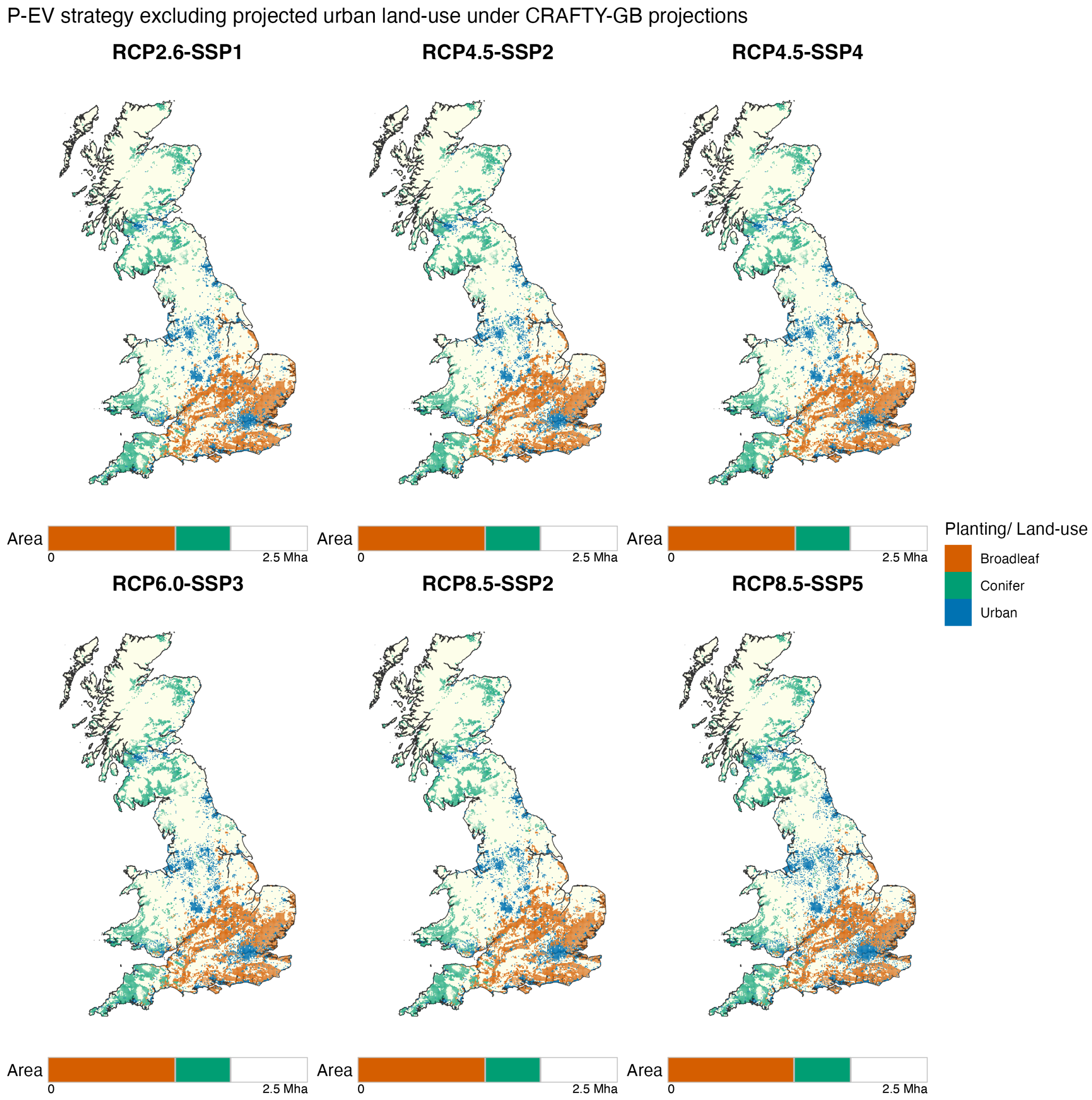

Fig. S6. P-EV strategies where grid cells with predicted future urban land development by 2050 (under the CRAFTY-GB agent-based land-use model) are excluded from tree planting, with the bars showing the mix of conifers and broadleaves, with bars indicating planting area (bar width: 2.5 million ha).

We also estimated the change in NPV of the planting strategy when areas of predicted future urban land development are excluded from planting, relative to a planting strategy where these areas are not excluded. These results were depicted in Table S3.

Land-use Projection	Change in NPV (Mean and 90% intervals, in billion £)
RCP2.6-SSP1	-0.9 (-5 – 2.54)
RCP4.5-SSP2	-0.9 (-5 – 2.53)
RCP4.5-SSP4	-0.89 (-4.98 – 2.55)
RCP6.0-SSP3	-0.91 (-5.01 – 2.52)
RCP8.5-SSP2	-0.9 (-5 – 2.53)
RCP8.5-SSP5	-1.02 (-5.07 – 2.4)

Table S3. Change in NPV (across all CERs) of the P-EV strategy after excluding future urban land-use according to CRAFTY-GB projections, compared to the P-EV strategy without exclusion of future urban land-use

In these maps, we show that the exclusion of land where future urban development is projected to occur results in qualitatively similar patterns to without that exclusion in terms of the mix of planting and the locations where planting occurred. This suggests that if the opportunity costs of urban development were accounted for, and even if those opportunity costs are dramatically higher than the benefits of tree planting (in those areas with future urban land-use), alternative planting locations that are outside of the areas with high urban development potential can still be found to meet Net Zero targets and do not strongly affect the net present value of tree-planting.

References

1. E. L. Robinson, C. Huntingford, V. S. Semeena, J. M. Bullock, CHESS-SCAPE: Future projections of meteorological variables at 1 km resolution for the United Kingdom 1980-2080 derived from UK Climate Projections 2018. NERC EDS Centre for Environmental Data Analysis. https://doi.org/10.5285/8194B416CBEE482B89E0DFBE17C5786C. Deposited 28 March 2022.
2. D. B. Lobell, W. Schlenker, J. Costa-Roberts, Climate trends and global crop production since 1980. *Science* **333**, 616–620 (2011).
3. D. B. Lobell, C. B. Field, Global scale climate–crop yield relationships and the impacts of recent warming. *Environ. Res. Lett.* **2**, 014002 (2007).
4. W. J. Sacks, D. Deryng, J. A. Foley, N. Ramankutty, Crop planting dates: an analysis of global patterns. *Glob. Ecol. Biogeogr.* **19**, 607–620 (2010).
5. C. Monfreda, N. Ramankutty, J. A. Foley, Farming the planet: 2. Geographic distribution of crop areas, yields, physiological types, and net primary production in the year 2000. *Global Biogeochem. Cycles* **22** (2008).
6. H. Lütkepohl, *New introduction to multiple time series analysis* (Springer Berlin Heidelberg, 2005).
7. A. R. Russell, G. C. van Kooten, J. G. Izett, M. E. Eiswerth, Damage Functions and the Social Cost of Carbon: Addressing Uncertainty in Estimating the Economic Consequences of Mitigating Climate Change. *Environ. Manage.* **69**, 919–936 (2022).
8. P. H. Howard, T. Sterner, Few and Not So Far Between: A Meta-analysis of Climate Damage Estimates. *Environ. Resour. Econ.* **68**, 197–225 (2017).
9. M. C. Hänsel, *et al.*, Climate economics support for the UN climate targets. *Nature Climate Change 2020 10:8* **10**, 781–789 (2020).
10. IIASA, Representative Concentration Pathways Database (RCP) | IIASA. (2010). Available at: https://iiasa.ac.at/models-and-data/representative-concentration-pathways-database.
11. B. Day, *et al.*, The Natural Environmental Valuation (NEV) Modelling Suite: A Summary Technical Report. (2020).
12. HM Treasury, *The Green Book: Central Government Guidance on Appraisal and Evaluation* (HM Treasury, 2020).
13. R. W. Thompson D. A and Matthews, “The storage of carbon in trees and timber” (Forestry Commission Research Information Note 160., 1989).
14. A. Daigneault, *et al.*, How the future of the global forest sink depends on timber demand, forest management, and carbon policies. *Glob. Environ. Change* **76**, 1–13 (2022).
15. A. Binner, *et al.*, “Natural Environment Valuation Online Tool Technical Documentation, Chapter 2: Forestry Model” (Land, Environment, Economics and Policy (LEEP) Institute University of Exeter, 2019).
16. I. J. Bateman, *et al.*, Spatially explicit integrated modeling and economic valuation of climate driven land use change and its indirect effects. *J. Environ. Manage.* (2016). https://doi.org/10.1016/j.jenvman.2016.06.020.
17. C. Fezzi, *et al.*, Valuing Provisioning Ecosystem Services in Agriculture: The Impact of Climate Change on Food Production in the United Kingdom. *Environ. Resour. Econ.* **57**, 197–214 (2014).
18. I. J. Bateman, *et al.*, Bringing ecosystem services into economic decision-making: Land use in the United Kingdom. *Science* **341**, 45–50 (2013).
19. P. D. L. Ritchie, *et al.*, Shifts in national land use and food production in Great Britain after a climate tipping point. *Nature Food* **1**, 76–83 (2020).
20. C. Brown, *et al.*, Agent-based modeling of alternative futures in the British land use system. *Earths Future* **10** (2022).
